# Cardiovascular toxicity associated with angiogenesis inhibitors: A comprehensive pharmacovigilance analysis based on the FDA Adverse Event Reporting System database from 2014 to 2021

**DOI:** 10.3389/fcvm.2022.988013

**Published:** 2022-10-13

**Authors:** YanFeng Wang, Chanjuan Cui, Xiayang Ren, Xinran Dong, Wei Cui

**Affiliations:** ^1^Department of Comprehensive Oncology, National Cancer Center/National Clinical Research Center for Cancer/Cancer Hospital, Chinese Academy of Medical Sciences and Peking Union Medical College, Beijing, China; ^2^Department of Laboratory Medicine, National Cancer Center/National Clinical Research Center for Cancer/Cancer Hospital, Chinese Academy of Medical Sciences and Peking Union Medical College, Beijing, China; ^3^Department of Pharmacy, National Cancer Center/National Clinical Research Center for Cancer/Cancer Hospital, Chinese Academy of Medical Sciences and Peking Union Medical College, Beijing, China; ^4^School of Electronics Engineering and Computer Science, Peking University, Beijing, China

**Keywords:** cardiovascular toxicity, angiogenesis inhibitors, FAERS database, real-world study, disproportionality analysis, pharmacovigilance analysis

## Abstract

**Background:**

The profiles of cardiovascular toxicity associated with angiogenesis inhibitors, including intravenous monoclonal antibodies (mAbs) and oral tyrosine kinase inhibitors (TKIs), targeting vascular endothelial growth factor (VEGF) remain poorly elucidated in real-world settings. This pharmacovigilance analysis aimed to comprehensively investigate the frequency, spectrum, timing, and outcomes of cardiovascular toxicities associated with angiogenesis inhibitors and to explore the differences in such patterns between mAbs and TKIs.

**Methods:**

Disproportionality analysis was performed by leveraging reports from the FDA Adverse Event Reporting System (FAERS) database from 2014 to 2021. Cardiovascular adverse events (AEs) were grouped into nine narrow categories using the Standardized Medical Dictionary for Regulatory Activities (MedDRA) Queries (SMQs). Reporting odds ratio (ROR) and information components (ICs) were calculated with statistical shrinkage transformation formulas and a lower limit of 95% confidence interval (CI) for ROR (ROR_025_) > 1 or IC (IC_025_) > 0, with at least three reports being considered statistically significant.

**Results:**

A total of 757,577 reports of angiogenesis inhibitors and 70,668 (9.3%) reports of cardiovascular AEs were extracted. Significant disproportionality was detected in angiogenesis inhibitors for cardiovascular AEs (IC_025_/ROR_025_ = 0.35/1.27). Bevacizumab (31.8%), a mAb, presented the largest number of reports, followed by sunitinib (12.4%), a TKI. Hypertension (SMQ) was detected with the strongest signal value (IC_025_/ROR_025_ = 1.73/3.33), followed by embolic and thrombotic events (SMQ) (IC_025_/ROR_025_ = 0.32/1.26). Hypertension showed the shortest time to onset with a median (interquartile range) value of 23 (8, 69) days, while embolic and thrombotic events had the longest value of 51 (16, 153) days. Notably, hypertension presented the lowest proportions of death and life-threatening events (10.9%), whereas embolic and thrombotic events posed the highest (29.3%). Furthermore, both mAbs (IC_025_/ROR_025_ = 0.47/1.39) and TKIs (IC_025_/ROR_025_ = 0.30/1.23) showed increased cardiovascular AEs. Hypertension was detected in both agents (IC_025_/ROR_025_ = 1.53/2.90 for mAbs and IC_025_/ROR_025_ = 1.83/3.56 for TKIs) with a shorter time to onset of 17 (6, 48) days for TKIs than mAbs of 42 (14, 131) days. By contrast, embolic and thrombotic events were detected for mAbs (IC_025_/ROR_025_ = 0.90/1.87) without TKI (IC_025_/ROR_025_ = −0.08/0.95).

**Conclusion:**

Angiogenesis inhibitors were associated with increased cardiovascular toxicity with a discrepancy between intravenous mAbs and oral TKIs, deserving distinct monitoring and appropriate management.

## Introduction

Angiogenesis plays a critical role in tumor growth and metastasis, and vascular endothelial growth factor (VEGF) has been confirmed to be the main proangiogenetic factor (1, 2). Targeting VEGF-induced angiogenesis to establish an anti-neoplastic effect was first proposed by Folkman in 1971 (3). Since bevacizumab, an anti-VEGF monoclonal antibody (mAb), was first approved in 2004 by the U.S. Food and Drug Administration (FDA) for the treatment of metastatic colorectal carcinoma in combination with chemotherapy (4), four main classes of agents targeting the VEGF signaling pathway have been developed: anti-VEGF mAb, anti-VEGF receptor (VEGFR) mAb, VEGF soluble decoy receptor capturing free available VEGF (VEGF-trap), and tyrosine kinase inhibitors (TKIs), that is, oral small-molecule agents that act on the intracellular tyrosine kinase domains of VEGFRs to inhibit their activation (1, 2). In contrast to mAbs, small-molecule TKIs target multiple tyrosine kinases other than VEGFs. In addition, recombinant human endostatin is another agent with an antiangiogenic effect, which was developed mainly in China (5).

Despite the remarkable anti-tumor effects of angiogenesis inhibitors in a variety of cancer cases, emerging evidence has shown cardiovascular toxicity associated with angiogenesis inhibitors (6–8). Although hypertension has received the most attention, a wider range of cardiovascular toxicity, including left ventricular systolic dysfunction, heart failure, myocardial ischemia, thromboembolic events, QT interval prolongation, and arrhythmia, has also been increasingly recognized (1, 2, 6–20). However, the majority of these data were from clinical trials, conducted in selected populations, which may underestimate the real burden of cardiovascular toxicity. Moreover, it is unclear whether the differences in the mechanism of action and route of administering between intravenous mAbs (including anti-VEGF mAb, anti-VEGFR mAb, and VEGF-trap) and oral TKIs with anti-VEGF(R) activity translate into clinically relevant differences in the incidence of cardiovascular toxicity.

Therefore, this pharmacovigilance analysis aimed to systematically investigate real-world patterns of total and class-specific cardiovascular toxicity associated with angiogenesis inhibitors and to explore the potential differences in such profiles between mAbs and TKIs with anti-VEGF(R) activity.

## Materials and methods

### Data sources

The U.S. FDA Adverse Event Reporting System (FAERS) database is a free post-marketing safety surveillance database that contains millions of real-world spontaneous adverse event (AE) reports submitted by healthcare professionals, individual patients, and drug manufacturers around the world (21). The large quantity of data collected at a national level from a large population and under conditions that may have been overlooked in controlled clinical trials makes FAERS particularly robust to conduct a pharmacovigilance study in the real-world setting.

In FAERS, AEs are coded using preferred terms (PTs) according to the Medical Dictionary for Regulatory Activities (MedDRA) (version 24.0). A specific PT can be assigned to several high-level terms (HLTs), high-level group terms (HLGTs), and system organ classes (SOCs). In addition, all PTs representing symptoms, signs, investigations, or diagnoses likely to be relevant can be grouped into meaningful categories using the Standardized MedDRA Queries (SMQs) to define a medical condition of interest. In this study, cardiovascular AEs were grouped into nine narrow categories of SMQs (cardiac arrhythmia, cardiac failure, cardiomyopathy, embolic and thrombotic events, hypertension, ischemic heart disease, noninfectious myocarditis/pericarditis, pulmonary hypertension, and torsade de pointes/QT prolongation) (Table 1) (see Supplementary Tables S1–S9) (22).

**Table 1:** Cardiovascular adverse events grouped into 9 narrow categories of Standardized MedDRA Queries (SMQs) according to MedDRA 24.0.

**SMQ name**	**SMQ code**	**Algorithm**
Cardiac arrhythmias	20000049	Narrow
Cardiac failure	20000004	Narrow
Cardiomyopathy	20000150	Narrow
Embolic and thrombotic events	20000081	Narrow
Hypertension	20000147	Narrow
Ischaemic heart disease	20000043	Narrow
Noninfectious myocarditis/pericarditis	20000239	Narrow
pulmonary hypertension	20000130	Narrow
torsade de pointes/QT prolongation	20000001	Narrow

### Data extract

This retrospective analysis enrolled data in the FAERS database from the first quarter of 2014 to the fourth quarter of 2021. Of note, there are inevitably duplicates (the same report submitted by different sources) and multiple reports (a follow-up of the same case with additional and updated information) in the spontaneous reporting database. Therefore, a two-step data cleaning was conducted before analysis. First, as for the reports with the same “safetyreportid,” only the last version of the reports was used. Second, reports with the same variables, such as “patientsex,” “patientonsetage,” “reportercountry,” “receiptdate,” “reaction meddra pt,” and “medicinal product,” were considered duplicated and removed. Furthermore, since time to onset was defined as the period between the start date of angiogenesis inhibitors and the onset date of cardiovascular AEs, reports without any information on the “drug start date” or “case event date” or start date of the drug later than the onset date of AEs were regarded as aberrant and excluded from the analysis of time to onset.

Notably, the drugs are reported as free text in FAERS, either generic names or brand names even research codes can be reported; and misspelling can also be present. Thus, a thorough drug name archive including all generic names, brand names, and research codes of angiogenesis inhibitors approved by the U.S. FDA or the National Medical Products Administration (NMPA) in China (formerly known as the China Food and Drug Administration, CFDA) was applied (see Supplementary Table S10).

### Statistical analysis

Currently, disproportionality analysis (also known as case–noncase analysis) is a widely used signal detection method in the pharmacovigilance study based on a two-by-two contingency table (Table 2) (23, 24).

**Table 2:** Disproportionality analysis based on two-by-two contingency table.

	**Target adverse events**	**Other adverse events**	**Total**
Target drug	a (N_observed_)	b	N_drug_=a+b
Other drugs	c	d	c+d
Total	N_event_=a+c	b+d	N_total_=a+b+c+d

Reporting odds ratio (ROR) and information components (ICs) are two specific indices calculated to detect potential associations between drugs and AEs. Notably, statistical shrinkage transformation was applied to obtain robust results, and the corresponding calculation formulas for ROR and IC are as follows (25):


$$\begin{array}{c} \text{ROR }=\;\left({\text{N}}_{\text{observed}}\;+\;0.5\right)/\left({\text{N}}_{\text{expected}}\;+\;0.5\right)\\ \text{IC }=\text{ lo}{\text{g}}_{2}\left[\left({\text{N}}_{\text{observed}}\;+\;0.5\right)/\left({\text{N}}_{\text{expected}}\;+\;0.5\right)\right]\\ {\text{N}}_{\text{expected}}\;=\;{\text{N}}_{\text{drug}}\;*\;{\text{N}}_{\text{event}}/{\text{N}}_{\text{total}}, \end{array}$$

where N_observed_ (a) is the observed number of reports of target drug AEs, N_expected_ is the expected number of reports of target drug AEs, N_drug_ (a+b) is the total number of reports of target drug, N_event_ (a+c) is the total number of reports of target AEs, and N_total_ (a+b+c+d) is the total number of reports in the whole database.

Moreover, the calculation formulas for the 95% confidence interval (CI) of the ROR and IC are as follows:


$$\begin{array}{c} \text{ROR }95\%\text{CI }=\;{\text{e}}^{\text{ln}\left(\text{ROR}\right)\;\pm \;1.96\sqrt{\frac{1}{\text{a}}\;+\;\frac{1}{\text{b}}\;+\;\frac{1}{\text{c}}\;+\;\frac{1}{\text{d}}}}\\ \text{I}{\text{C}}_{025}\;=\text{ IC }-\;3.3^{*}{\left({\text{N}}_{\text{observed}}\;+\;0.5\right)}^{-0.5}\\ -2{\;}^{*}\;{\left({\text{N}}_{\text{observed}}\;+\;0.5\right)}^{-1.5}\\ \text{I}{\text{C}}_{075}\;=\text{ IC }+\;2.4^{*}{\left({\text{N}}_{\text{observed}}\;+\;0.5\right)}^{-0.5}\\ -0.5^{*}\;{\left({\text{N}}_{\text{observed}}\;+\;0.5\right)}^{-1.5} \end{array}$$

The lower limit of the 95% CI for ROR (ROR_025_) >1 or the lower limit of the 95% CI for IC (IC_025_) exceeding 0 with at least three reports was considered statistically significant and deemed a potential signal.

All the analyses were performed using SAS version 9.4 (SAS Institute Inc., Cary, NC, United States).

## Results

### Descriptive analysis

**Figure 1:**
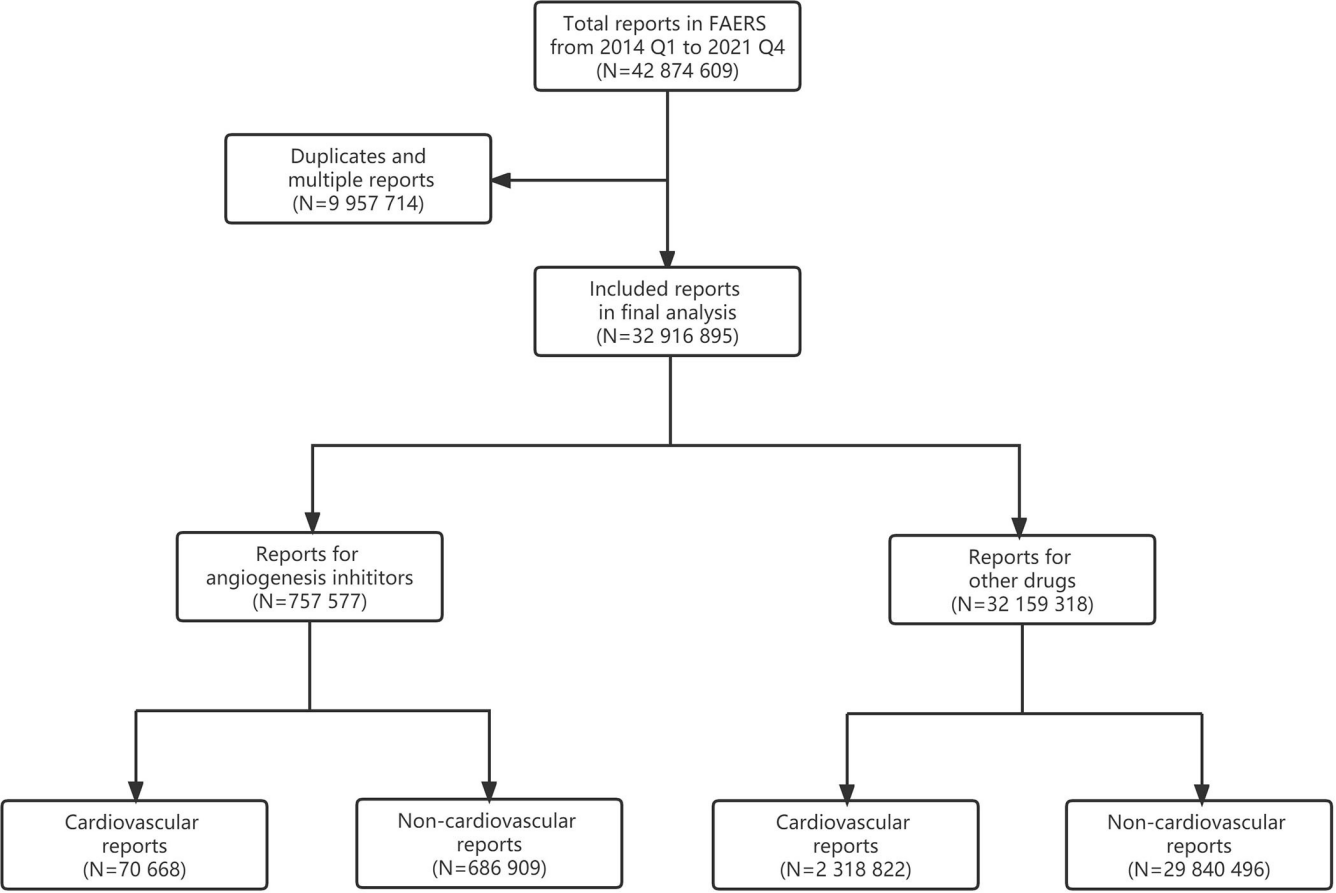
Flowchart of report selection.

From the first quarter of 2014 to fourth quarter of 2021, a total of 42,874,609 reports were extracted from the FAERS database and 32,916,895 reports were included in the final analysis, of which 757,577 reports on angiogenesis inhibitors and 70,668 (9.3%) reports of cardiovascular AEs were extracted (Figure 1).

Characteristics of cardiovascular AE reports are presented in Table 3. The median [interquartile range (IQR)] age of patients with angiogenesis inhibitors was 66 (57, 73) years, which was older than those of 61 (47, 72) years with other drugs. Among cardiovascular reports associated with angiogenesis inhibitors, older patients (aged of ≥ 65 years vs. 18–64 years: 40.5% vs. 33.1%) and male patients (49.1% vs. 41.3%) accounted for a greater proportion than younger patients and female patients, respectively. In addition, cardiovascular AEs with angiogenesis inhibitors were chiefly submitted by health professionals (65.0%) and mainly from the United States (46.3%). As for the outcomes of AEs, caused or prolonged hospitalization, other serious events and death were the most frequently reported.

**Table 3:** Baseline characteristics of cardiovascular reports associated with angiogenesis inhibitors and other drugs from 2014 to 2021.

**Characteristics**	**Angiogenesis inhibitors** **(*n* = 70 668)**	**Other drugs** **(*n* = 2 318 822)**	**Total** **(*n* = 2 389 490)**
Patient's age, years, median (Q1-Q3)	66 (57, 73)	61 (47, 72)	61 (48, 72)
Data available, *n* (%)	52,859 (74.8)	1,603,471 (69.2)	1,656,882 (69.3)
**Age group**, ***n*** **(%)**			
<18 years	847 (1.2)	62,446 (2.7)	63,293 (2.7)
18~65 years	23,401 (33.1)	863,660 (37.3)	887,061 (37.1)
≥ 65 years	28,611 (40.5)	677,365 (29.2)	706,528 (29.6)
Unknown	17,809 (25.2)	715,351 (30.8)	732,608 (30.7)
**Patient's gender**, ***n*** **(%)**			
Male	34,731 (49.1)	886,247 (38.2)	920,978 (38.6)
Female	29,183 (41.3)	1,223,706 (52.8)	1,252,889 (52.4)
Unknown	6,754 (9.6)	208,869 (9.0)	215,623 (9.0)
**Type of reporter**, ***n*** **(%)**			
Health professional	45,906 (65.0)	1,278 704 (55.2)	1,324,610 (55.4)
Non-health professional	23,572 (33.3)	972,435 (41.9)	996,007 (41.7)
Unknown	1,190 (1.7)	67,683 (2.9)	68,873 (2.9)
**Outcome of adverse events**, ***n*** **(%)**			
Death	13,300 (18.8)	286,691 (12.4)	299,991 (12.6)
Life-threatening	3,117 (4.4)	141,776 (6.1)	144,893 (6.1)
Caused/prolonged hospitalization	24,975 (35.3)	816,713 (35.2)	841,688 (35.2)
Disabling/incapacitating	384 (0.5)	29,124 (1.2)	29,508 (1.2)
Congenital anomaly	1 (0)	4,041 (0.2)	4,042 (0.2)
Other serious events	19,391 (27.4)	664,311 (28.6)	683,702 (28.6)
**Reported countries**, ***n*** **(%)**			
United States	32,755 (46.3)	1,242,146 (53.6)	1,274,901 (53.4)
Canada	3,606 (5.1)	189,636 (8.2)	193,242 (8.1)
Great Britain	1,915 (2.7)	100,385 (4.3)	102,300 (4.3)
Germany	3,404 (4.8)	93,682 (4.0)	97,086 (4.1)
France	3,611 (5.1)	88,021 (3.8)	91,632 (3.8)
Italy	2,094 (3.0)	53,081 (2.3)	55,175 (2.3)
Japan	7,085 (10.0)	79,696 (3.4)	86,781 (3.6)
China	1,610 (2.3)	21,911 (0.9)	23,521 (1.0)
Other countries	12,519 (17.8)	355,163 (15.4)	367,682 (15.4)
Unknown	2,069 (2.9)	95,101 (4.1)	97,170 (4.0)
**Reported year**, ***n*** **(%)**			
2014	7,966 (11.3)	296,750 (12.8)	304,716 (12.8)
2015	9,418 (13.3)	357,180 (15.4)	366,598 (15.3)
2016	6,392 (9.1)	221,264 (9.6)	227,656 (9.5)
2017	7,076 (10.0)	229,409 (9.9)	236,485 (9.9)
2018	9,587 (13.6)	287,918 (12.4)	297,505 (12.5)
2019	8,917 (12.6)	271,430 (11.7)	280,347 (11.7)
2020	9,548 (13.5)	290,083 (12.5)	299,631 (12.5)
2021	11,764 (16.6)	364,788 (15.7)	376,552 (15.8)

### Disproportionality analysis of cardiovascular AEs for angiogenesis inhibitors

Of note, most cardiovascular AEs were reported in cases using TKIs (*N* = 45 475, 62.4%), among which sunitinib was the most common reported agent (*N* = 9 061, 12.4%). By contrast, bevacizumab (*N* = 23 177, 31.8%), an anti-VEGF mAb, presented the largest number of reported AEs as a single agent (Table 4).

**Table 4:** Disproportionality analysis results associated with different angiogenesis inhibitors.

**Drug class**	**Agent**	***N* (%)**	**ROR**	**ROR025**	**ROR975**	**IC**	**IC025**	**IC975**
Anti-VEGF mAb	Bevacizumab	23,177 (31.8)	1.37	1.35	1.39	0.45	0.43	0.47
Anti-VEGFR mAb	Ramucirumab	1,193 (1.6)	1.49	1.40	1.58	0.57	0.48	0.64
VEGF-Trap	Aflibercept	1,596 (2.2)	1.71	1.62	1.80	0.77	0.69	0.83
Tyrosine kinase inhibitors	Sunitinib	9,061 (12.4)	1.06	1.04	1.08	0.08	0.05	0.11
	Lenvatinib	7,131 (9.8)	1.74	1.70	1.79	0.80	0.76	0.83
	Nintedanib	6,824 (9.4)	1.40	1.36	1.43	0.48	0.44	0.51
	Pazopanib	6,277 (8.6)	1.25	1.22	1.28	0.32	0.28	0.35
	Cabozantinib	4,827 (6.7)	1.05	1.02	1.08	0.07	0.02	0.10
	Sorafenib	4,473 (6.1)	1.13	1.10	1.16	0.18	0.13	0.21
	Axitinib	3,402 (4.7)	1.36	1.31	1.40	0.44	0.38	0.48
	Regorafenib	3,184 (4.4)	1.04	1.01	1.08	0.06	0.01	0.10
	Apatinib (China)	947 (1.3)	1.23	1.15	1.31	0.30	0.19	0.37
	Vandetanib	487 (0.7)	1.48	1.35	1.63	0.57	0.42	0.68
	Tivozanib	104 (0.1)	1.42	1.16	1.74	0.51	0.18	0.74
	Cediranib	71	2.60	2.01	3.37	1.38	0.98	1.66
	Erdafitinib	45	0.47	0.35	0.64	−1.08	−1.57	−0.72
	Fruquintinib (China)	5	0.75	0.30	1.85	−0.41	−1.98	0.57
	Vatalanib	2	N	N	N	N	N	N
	Anlotinib (China)	1	N	N	N	N	N	N
	All TKIs	45,475 (62.4)	1.24	1.23	1.26	0.32	0.30	0.33
Other	Recombinant human endostatin (China)	26	1.34	0.89	2.00	0.42	−0.24	0.88
Total		70,668	1.29	1.27	1.30	0.36	0.35	0.37

VEGF, vascular endothelial growth factor; mAb, monoclonal antibody; VEGFR, vascular endothelial growth factor receptor, TKI, tyrosine kinase inhibitors; ROR, reporting odds ratio; IC, information components.

Using angiogenesis inhibitors was significantly associated with a higher reporting frequency of cardiovascular AEs than the whole database corresponding to an ROR (ROR_025_, ROR_975_) of 1.29 (1.27, 1.30) and an IC (IC_025_, IC_975_) of 0.36 (0.35, 0.37) (Table 4).

Notably, significant signals were detected in the majority of agents, except for erdafitinib, fruquintinib (China), vatalanib, anlotinib (China), and recombinant human endostatin (China). Since these agents accounted for a very small proportion of AEs reported with no significant signals detected as a consequence, these agents were not included in the further analysis as single agents.

As for the signal strength, TKIs as a class of agents demonstrated the weakest signal value (IC_025_/ROR_025_ = 0.30/1.23) compared with anti-VEGF mAb (IC_025_/ROR_025_ = 0.43/1.35), anti-VEGFR mAb (IC_025_/ROR_025_ = 0.48/1.40), and VEGF-Trap (IC_025_/ROR_025_ = 0.69/1.62).

In addition, with respect to single agent, cediranib held the strongest signal value (IC_025_/ROR_025_ = 0.98/2.01), despite a very small proportion reported (*N* = 71, < 0.1%), while regorafenib (*N* = 3 184, 4.4%) showed the weakest signal value (IC_025_/ROR_025_ = 0.01/1.01).

### Spectrum of cardiovascular AEs based on PTs for angiogenesis inhibitors

Overall, hypertension (*N* = 10 654, 15.1%) contributed to the most frequently reported cardiovascular PTs associated with angiogenesis inhibitors, followed by dyspnea (*N* = 7 739, 11.0%) and increased blood pressure (*N* = 6 404, 9.1%).

According to IC_025_ >0, a total of 112 PTs were observed to be significantly associated with angiogenesis inhibitors as a whole. For single agent, bevacizumab presented the broadest spectrum of cardiovascular AEs with a total of 106 PTs detected as signals, while cediranib held the least PTs (*N* = 3) (see Supplementary Table S11).

Of note, hypertension was detected as signals in 13 agents, except apatinib (China) and tivozanib, which were the most frequently reported PTs. Furthermore, blood pressure increased, ejection fraction decreased, and ascites with another three PTs detected as signals among 11 agents (see Supplementary Table S12).

### Spectrum of cardiovascular AEs based on SMQs for angiogenesis inhibitors

As seen in Table 5, among the nine narrow categories of SMQs, cardiomyopathy (SMQ) (*N* = 22 186, 22.7%) comprised the most frequently reported cardiovascular AEs, followed by hypertension (SMQ) (*N* = 19 385, 19.8%) and embolic and thrombotic events (SMQ) (*N* = 15 365, 15.7%).

**Table 5:** Disproportionality analysis for angiogenesis inhibitors based on specific SMQs.

**Cardiovascular reports**	***N* (%)**	**ROR**	**ROR025**	**ROR975**	**IC**	**IC025**	**IC975**
Cardiac arrhythmias (SMQ)	8,607 (8.8)	0.65	0.63	0.66	−0.63	−0.67	−0.60
Cardiac failure (SMQ)	9,031 (9.2)	1.12	1.10	1.14	0.16	0.13	0.19
Cardiomyopathy (SMQ)	22,186 (22.7)	1.10	1.09	1.12	0.14	0.12	0.15
Embolic and thrombotic events (SMQ)	15,365 (15.7)	1.28	1.26	1.30	0.35	0.32	0.37
Hypertension (SMQ)	19,385 (19.8)	3.38	3.33	3.43	1.76	1.73	1.78
Ischaemic heart disease (SMQ)	3,672 (3.7)	0.79	0.76	0.82	−0.34	−0.40	−0.30
Noninfectious myocarditis/pericarditis (SMQ)	5,849 (6.0)	1.01	0.98	1.03	0.01	−0.03	0.04
Pulmonary hypertension (SMQ)	10,009 (10.2)	1.10	1.08	1.12	0.14	0.11	0.16
Torsade de pointes/QT prolongation (SMQ)	3,524 (3.6)	0.68	0.66	0.71	−0.55	−0.60	−0.51

SMQ, standardized MedDRA queries; ROR, reporting odds ratio; IC, information components.

Specifically, hypertension (SMQ) held the strongest signal value (IC_025_/ROR_025_ = 1.73/3.33), followed by embolic and thrombotic events (SMQ) (IC_025_/ROR_025_ = 0.32/1.26), cardiac failure (SMQ) (IC_025_/ROR_025_ = 0.13/1.10), cardiomyopathy (SMQ) (IC_025_/ROR_025_ = 0.12/1.09), and pulmonary hypertension (SMQ) (IC_025_/ROR_025_ = 0.11/1.08). However, cardiac arrhythmias (SMQ), ischemic heart disease (SMQ), noninfectious myocarditis/pericarditis (SMQ), and torsade de pointes/QT prolongation (SMQ) were not observed as significantly associated with angiogenesis inhibitors as a whole.

Based on MedDRA, embolic and thrombotic events (SMQ) can be subcategorized into embolic and thrombotic events, arterial thromboembolic events (ATEs) (SMQ), embolic and thrombotic events, venous thromboembolic events (VTEs) (SMQ), and embolic and thrombotic events, vessel type unspecified, and mixed arterial and venous (SMQ). Further analysis showed that both ATEs (IC_025_/ROR_025_ = 0.01/1.01) and VTEs (IC_025_/ROR_025_ = 1.03/2.06) were significantly associated with angiogenesis inhibitors.

**Figure 2:**
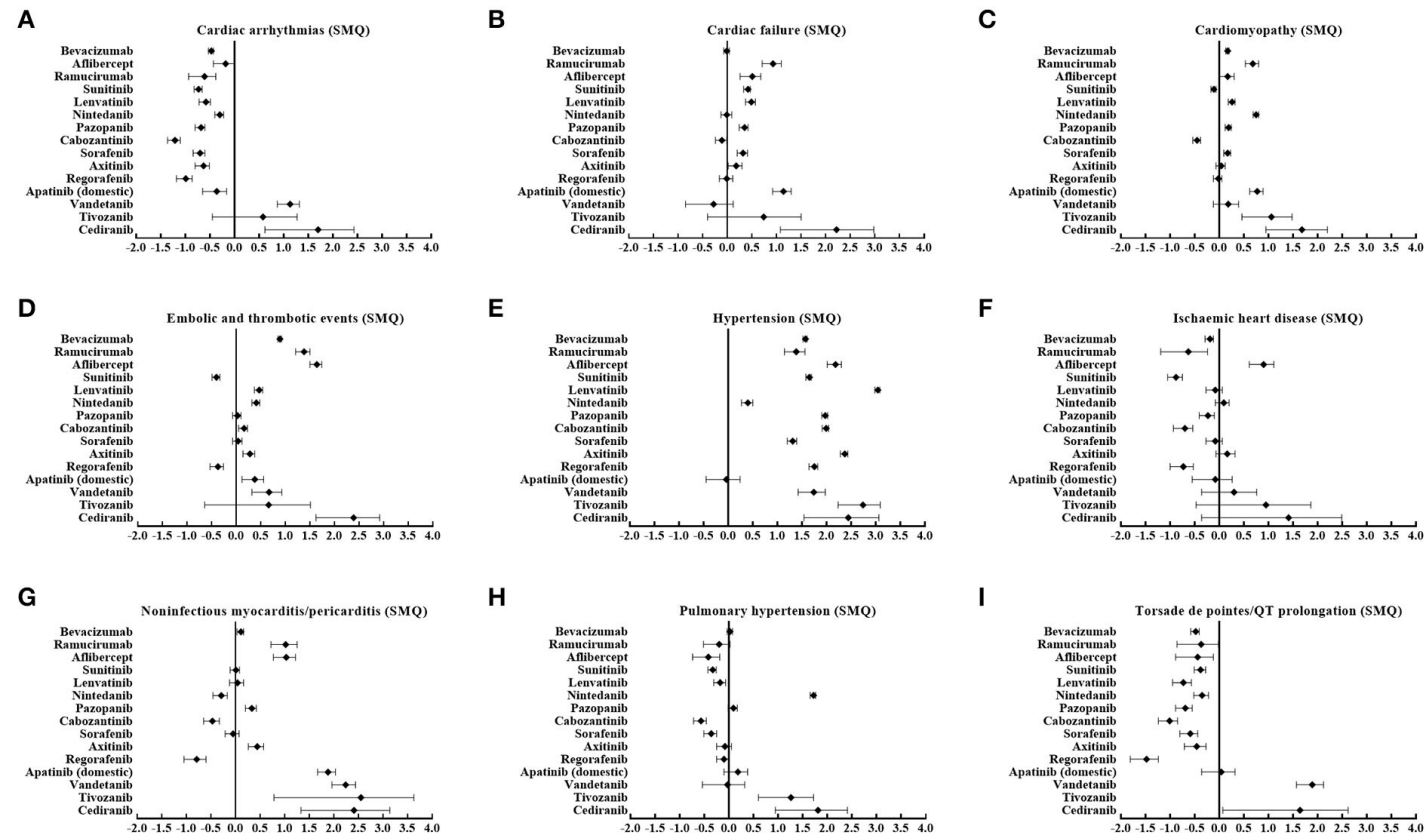
**(A–I)** Cardiovascular toxicity profiles based on nine narrow categories of (SMQs) according to ICs among different angiogenesis inhibitors. SMQs, Standardized MedDRA Queries; IC, information components.

Importantly, analysis based on a single agent showed varied patterns of cardiovascular AEs among different angiogenesis inhibitors, as depicted in Figure 2. Of note, aflibercept was the only agent significantly associated with ischemic heart disease (SMQ) (IC_025_ = 0.61). Furthermore, cardiac arrhythmias (SMQ) were observed to be significantly associated with vandetanib (IC_025_ = 0.87) and cediranib (IC_025_ = 0.62). Similarly, torsade de pointes/QT prolongation (SMQ) is also associated with vandetanib (IC_025_ = 1.57) and cediranib (IC_025_ = 0.07). In addition, nintedanib (IC_025_ = 1.65), tivozanib (IC_025_ = 0.60), and cediranib (IC_025_ = 0.94) were the only three agents related to pulmonary hypertension (SMQ). By contrast, cardiac failure (SMQ), cardiomyopathy (SMQ), and noninfectious myocarditis/pericarditis (SMQ) were detected as signals in nine agents, embolic and thrombotic events (SMQ) was detected in 10 agents, and hypertension (SMQ) was detected as signals in 14 agents, except apatinib (China), which was the most frequently reported PTs.

### Time to onset of specific SMQs with significant signals

**Figure 3:**
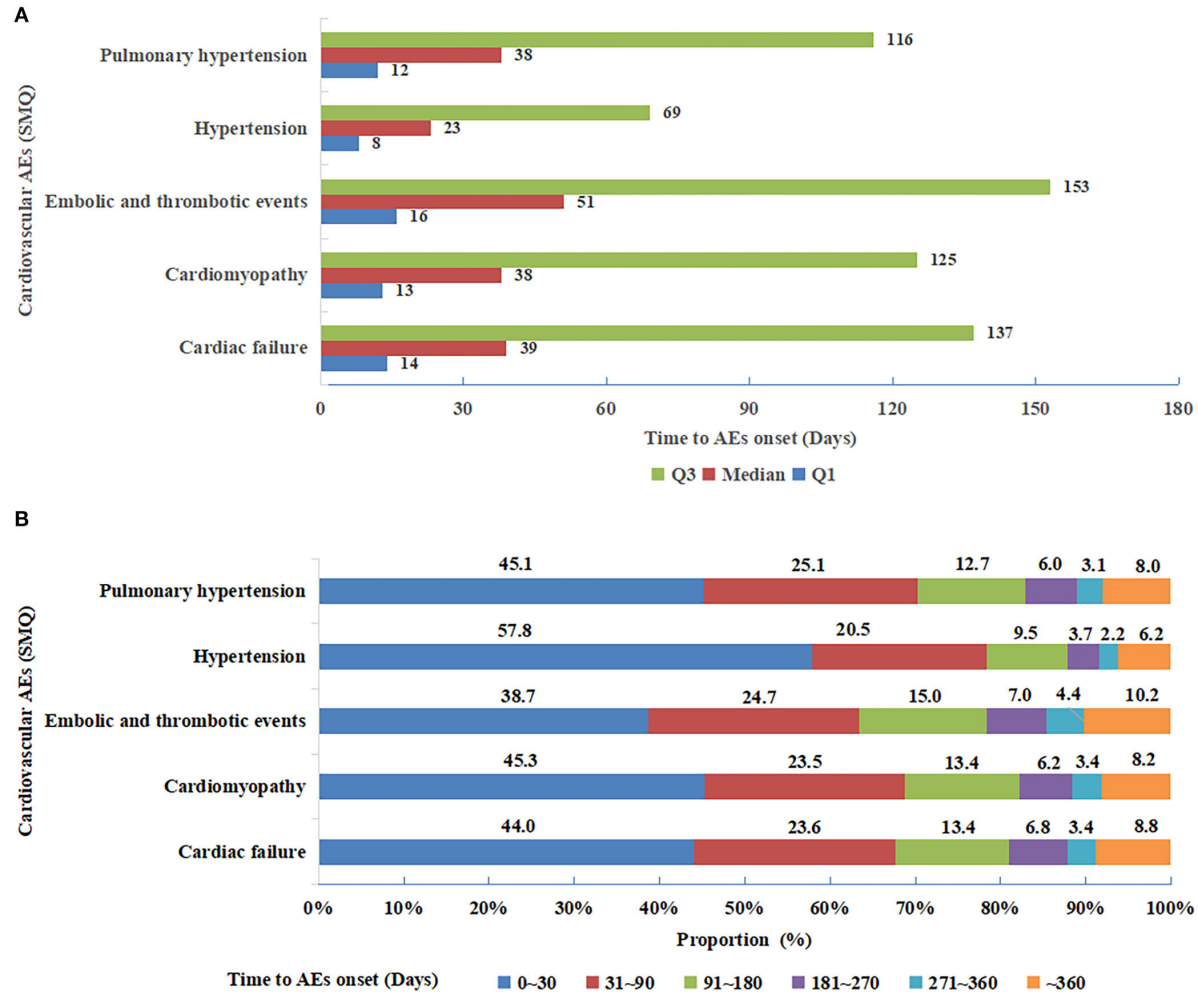
**(A)** Median (interquartile range) of time to onset for five cardiovascular adverse events (SMQs) detected as significant signals. SMQs, Standardized MedDRA Queries. **(B)** Time to onset groups for five cardiovascular adverse events (SMQs) detected as significant signals. SMQs, Standardized MedDRA Queries.

As displayed in Figure 3A, hypertension (SMQ) demonstrated the shortest time to onset with the median (IQR) value of 23 (8, 69) days, while embolic and thrombotic events (SMQ) had the longest time to onset of 51 (16, 153) days. Nonetheless, cardiac failure (SMQ), cardiomyopathy (SMQ), and pulmonary hypertension (SMQ) presented similar median values (IQR) of time to onset. Furthermore, the cumulative proportions of time to onset within the first 30 days and 90 days after treatment with angiogenesis inhibitors were 57.8% and 78.3% for hypertension (SMQ), which was the greatest, whereas they were 38.7% and 63.4% for embolic and thrombotic events (SMQ), which was the lowest (Figure 3B).

### Outcome of adverse events

**Figure 4:**
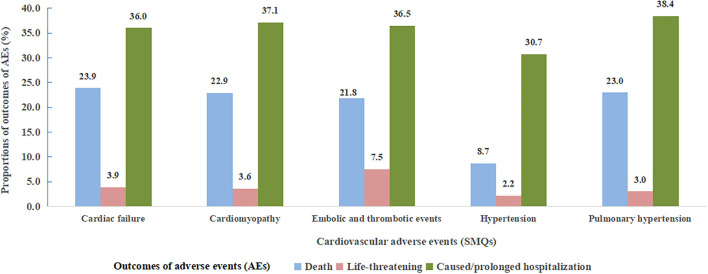
Proportions of cardiovascular adverse event outcomes according to five cardiovascular adverse events (SMQs) detected as significant signals. SMQs, Standardized MedDRA Queries; AEs, adverse events.

Of note, death accounted for 18.8% of all cardiovascular AEs associated with angiogenesis inhibitors, which was higher than that with other drugs (12.4%) (Table 3). Specifically, higher risk level outcomes, namely, death, life-threatening events, and caused or prolonged hospitalization proportions according to specific SMQs with significant signals are shown in Figure 4. Notably, hypertension (SMQ) presented the lowest proportions of death and life-threatening events (10.9%) and caused or prolonged hospitalization (30.7%), whereas embolic and thrombotic events (SMQ) posed the highest proportions of death and life-threatening events (29.3%) and a similar proportion of caused or prolonged hospitalization (36.5%) compared with cardiac failure (SMQ), cardiomyopathy (SMQ), and pulmonary hypertension (SMQ).

### Comparison of cardiovascular AEs between intravenous mAbs and oral TKIs with anti-VEGF(R) activity

Both mAbs and TKIs with anti-VEGF(R) activity were significantly associated with an increased risk of cardiovascular AEs, with a relatively stronger signal strength for mAbs (IC_025_/ROR_025_ = 0.47/1.39) than TKIs (IC_025_/ROR_025_ = 0.30/1.23).

As for PTs, Supplementary Table S13 showed the top 20 PTs with the strongest signal values between mAbs and TKIs. There was a great difference in the distribution of these PTs with only six PTs overlapping between these two classes with consistently stronger signals for mAbs.

**Figure 5:**
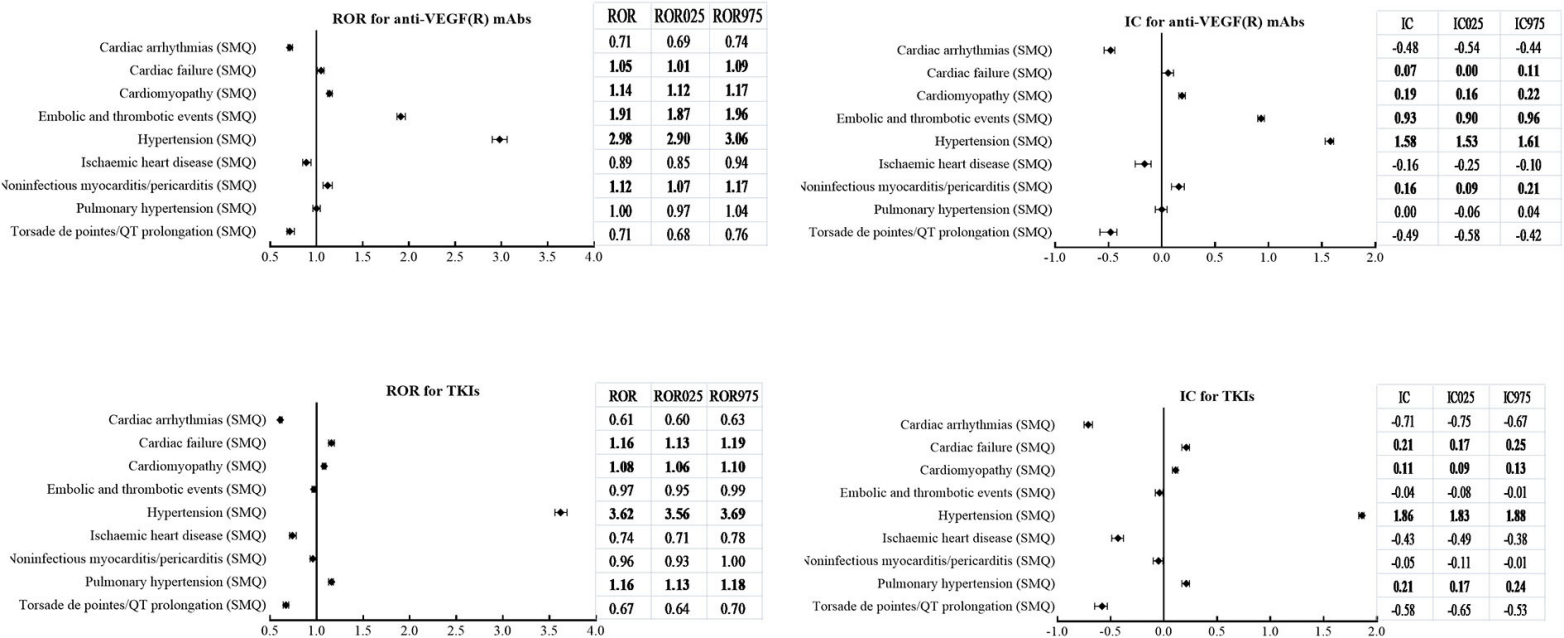
Comparison of cardiovascular adverse events (SMQs) according to ROR (ROR_025_, ROR_975_) and IC (IC_025_, IC_975_) between mAbs and TKIs with anti-VEGF(R) effects. SMQs, Standardized MedDRA Queries; ROR, reporting odds ratio; ROR_025_, lower limit of ROR; ROR_975_, upper limit of ROR; IC, information components; IC_025_, lower limit of IC; IC_975_, upper limit of IC; mAbs, monoclonal antibodies; TKIs, tyrosine kinase inhibitors; VEGF(R), vascular endothelial growth factor or receptor.

According to SMQs, Figure 5 demonstrates that significant signals were detected in hypertension (SMQ), cardiac failure (SMQ), and cardiomyopathy (SMQ) for both classes, embolic and thrombotic events (SMQ) and noninfectious myocarditis/pericarditis (SMQ) only for mAbs, and pulmonary hypertension (SMQ) only for TKIs. However, cardiac arrhythmias (SMQ), ischemic heart disease (SMQ), and torsade de pointes/QT prolongation (SMQ) were not detected as signals in both classes.

With respect to hypertension (SMQ), analysis of timing revealed a remarkably shorter time to onset for TKIs than mAbs, with a median (IQR) value of 17 (6, 48) days vs. 42 (14, 131) days. In addition, the cumulative proportions of time to onset within the first 30 days and 90 days were 65.8 and 84.7% for TKIs, which were higher than those of 42.0 and 65.8% for mAbs.

With regard to embolic and thrombotic events (SMQ), a total of 59 PTs were detected to be significantly associated with mAbs, whereas 23 PTs were found to be related to TKIs. Furthermore, 16 PTs overlapped between mAbs and TKIs, and the majority with stronger signal values (IC_025_) for mAbs except for two PTs with a stronger signal strength for TKIs (see Supplementary Table S14) were observed.

Both ATEs (IC_025_/ROR_025_ = 0.53/1.46) and VTEs (IC_025_/ROR_025_ = 1.79/3.47) held a significantly higher reporting frequency for mAbs. On the contrary, the signal was detected only in VTEs (IC_025_/ROR_025_ = 0.80/1.76), and no signal was detected in ATEs (IC_025_/ROR_025_ = −0.12/0.93) for TKIs.

## Discussion

Although the application of angiogenesis inhibitors has revolutionized the therapy and substantially improved the outcomes for patients with a variety of malignancies, their side effects, especially cardiovascular toxicity, have been increasingly recognized along with their curative effects (1, 2, 6–20). Nevertheless, the real profiles of cardiovascular toxicity associated with angiogenesis inhibitors are still unclear due to scarce evidence in the real-world setting (20). To the best of our knowledge, this is the first comprehensive pharmacovigilance study on cardiovascular toxicity associated with angiogenesis inhibitors by leveraging the FAERS database, involving the frequency, spectrum, timing, and outcomes of cardiovascular toxicity, as well as the extensive comparison of such patterns between mAbs and TKIs with anti-VEGF(R) activity. Importantly, the main findings of our study are as follows.

### Cardiovascular toxicity burden and profile of angiogenesis inhibitors

First, our study revealed that cardiovascular reports accounted for a proportion of 9.3% of all reports related to angiogenesis inhibitors. Notably, using angiogenesis inhibitors was significantly associated with an increased risk of cardiovascular AEs according to the markedly higher signal values of IC_025_/ROR_025_ = 0.36/1.27. In addition, all the four main classes of agents targeting the VEGF signaling pathway and the majority of single agents demonstrated this trend, except for few agents, which may be due to a very small number of reported AEs. To sum up, these findings indicated that cardiovascular toxicity may be a class effect of angiogenesis inhibitors. Given its continuous development and wide application, the incidence of angiogenesis inhibitor-related cardiovascular toxicity is expected to increase constantly, which necessitates more attention to be paid.

Noteworthily, our study found that cardiovascular toxicity profiles varied among different agents of angiogenesis inhibitors, such as different agents and different AEs. On the one hand, regarding specific agent, bevacizumab was first approved for clinical use; accordingly, its cardiovascular toxicity has been first and extensively studied over the past years (10, 13). Similarly, bevacizumab presented the largest number of reported AEs with relatively stronger signal strength as a single agent and held the broadest spectrum of cardiovascular AEs in our study. On the contrary, despite a very small proportion reported, cediranib held the strongest signal value for total cardiovascular AEs and some specific cardiovascular AEs, including cardiac failure (SMQ), cardiomyopathy (SMQ), and embolic and thrombotic events (SMQ). On the other hand, as for specific cardiovascular AEs, hypertension, to date, is the most frequently reported and best characterized cardiovascular toxicity associated with angiogenesis inhibitors targeting VEGF (9, 16, 17, 26, 27). Similar to previous studies, the present study demonstrated that hypertension was the most frequently reported cardiovascular AE based on PTs (15.1%) and SMQs (19.8%). Moreover, hypertension was the most frequently detected signal among the agents observed (13/15), with the strongest signal value of IC_025_/ROR_025_ = 1.73/3.33 based on SMQs and a relatively higher value of IC_025_/ROR_025_ = 2.06/4.19 based on PTs.

Especially, as a single agent, lenvatinib presented the strongest signal strength for hypertension (SMQ) (IC_025_=2.98), indicating the most frequently reported PT, which was similar to a recent report (28) showing the highest incidence of any grade hypertension of 68% and grade 3 or 4 hypertension of 42% for lenvatinib. In addition, two meta-analyses revealed a higher incidence of torsade de pointes/QT prolongation for vandetanib (29, 30). Our present study demonstrated similar results.

Taken together, all these findings suggested a great discrepancy of cardiovascular AEs with specific agents, which deserves some individual AE monitoring strategies for angiogenesis inhibitors in the treatment of cancer types.

### Timing and outcomes of cardiovascular AEs with angiogenesis inhibitors

As for the time to onset of cardiovascular AEs, in Österlund's study (31), the median time to the onset of hypertension was 1 month (range of 1–15 months and within 6 months in 95%), as calculated from the start of bevacizumab treatment. In addition, a real-life study on the TKI cohort showed that the cumulative incidence of thrombotic events kept increasing all along the first year of treatment (20). Nevertheless, no study to date has investigated the disparity of timing according to different types of cardiovascular AEs associated with angiogenesis inhibitors. Our present study first compared the time to onset of specific cardiovascular AEs based on SMQs, which provided some valuable information. We found that hypertension occurred fairly early, whereas embolic and thrombotic events occurred relatively late. Of note, virtually almost every patient experiences a rapid increase in blood pressure within days after initiation of therapy (26), whether or not leading to hypertension. Therefore, recognition of the variance in time to onset among different cardiovascular AEs may be worthwhile at clinical practice to guide distinct monitoring strategies.

With regard to the outcomes of AEs, the present study showed that cardiovascular AEs associated with angiogenesis inhibitors presented more death than other drugs, suggesting a greater impact on patients' prognosis. In addition, we compared the differences in outcomes among specific cardiovascular AEs based on SMQs and found that hypertension posed the lowest risk of mortality, despite being most frequently reported in comparison to other cardiovascular AEs. It may be due to the less severity of hypertension *per se* as well as appropriate management with anti-hypertensive medications to some extent. However, the prognostic value of hypertension induced by angiogenesis inhibitors, namely, whether or not it was a biomarker for the efficacy of anti-cancer treatment, remains the subject of investigation (32–35). Furthermore, we found that embolic and thrombotic events (SMQ) posed the highest proportions of death and life-threatening events among the reported cardiovascular AEs (SMQs).

### Differences in cardiovascular AEs between intravenous mAbs and oral TKIs

To our knowledge, no head-to-head study so far has compared the differences in cardiovascular AEs between mAbs and TKIs with anti-VEGF(R) activity. A systematic review and meta-analysis showed no significant interaction between the two subgroups for cardiovascular outcomes (12). Noteworthily, our study made the foremost and extensive comparison between mAbs and TKIs and thus provided several new insights into cardiovascular AEs associated with angiogenesis inhibitors. First, both mAbs and TKIs as class agents significantly increased cardiovascular AE risk with greater extent for mAbs. Second, there was a discrepancy in the distribution of cardiovascular AEs based on PTs or SMQs. Third, for hypertension (SMQ), TKIs demonstrated a relatively stronger signal strength and remarkably shorter time to onset than mAbs. Last, we also observed the variance in embolic and thrombotic events (SMQ), including ATEs and VTEs. Explanations for these discrepancies may be multifarious, mainly including the following two aspects: (1) difference in action mechanism, namely, unlike mAbs with high affinity to VEGF(R) (on-target mechanism), TKIs typically target multiple tyrosine kinases other than VEGFs, which, consequently, may induce some “off-target” toxicities besides the “on-target” effects; (2) difference in the administering route, that is, mAb is usually administered intravenously, while TKI is used orally, which theoretically can generate some variances in AEs. Anyway, these findings again underscore the necessity to pay more attention to agent-specific AEs.

## Limitations

There are some limitations in this study to be acknowledged. First, as a spontaneous reporting database, the FAERS database has some intrinsic limitations, such as multiple data sources, non-uniform data format, multi-reporting, under-reporting, and incomplete information. Second, the causal relationship of AEs and drug application cannot be confirmed in the retrospective study. Third, it is difficult to evaluate the effect of patients' baseline characteristics including cardiovascular risk factor profiles on the subsequent occurrence of cardiovascular AEs after application of angiogenesis inhibitors since no relevant variables other than age and sex were reported in the FAERS database. Finally, the majority of the reporting data in the FAERS database are from North America (particularly the United States), European countries, and Japan, while few reports come from China, which might result in geographic bias of the results. Therefore, further prospective studies may be warranted to confirm the findings in our study.

## Conclusion

Treatment with angiogenesis inhibitors was significantly associated with an increased risk in cardiovascular toxicity as a class effect mainly involving cardiac failure, cardiomyopathy, hypertension, embolic and thrombotic events, and pulmonary hypertension with varied profiles in terms of frequency, spectrum, timing, and outcomes among specific agents and special AEs. Moreover, there was a great discrepancy in cardiovascular toxicity patterns between mAbs and TKIs with anti-VEGF(R) activity. These findings provide valuable evidence for precise management of cardiovascular toxicity associated with angiogenesis inhibitors in the treatment of cancer cases.

## Data availability statement

The raw data supporting the conclusions of this article will be made available by the authors, without undue reservation.

## Ethics statement

Ethical review and approval was not required for the study on human participants in accordance with the local legislation and institutional requirements. Written informed consent for participation was not required for this study in accordance with the national legislation and the institutional requirements.

## Author contributions

YW: conception, design, and manuscript reviewing and revising. WC: administrative support. XR and XD: data collection and analysis. YW and CC: data interpretation. CC: manuscript writing. All authors contributed to the article and approved the final manuscript.

## Funding

This study was funded by the program of Beijing Hope Run Special Fund of Cancer Foundation of China (LC2017A13).

## Conflict of interest

The authors declare that the research was conducted in the absence of any commercial or financial relationships that could be construed as a potential conflict of interest.

## Publisher's note

All claims expressed in this article are solely those of the authors and do not necessarily represent those of their affiliated organizations, or those of the publisher, the editors and the reviewers. Any product that may be evaluated in this article, or claim that may be made by its manufacturer, is not guaranteed or endorsed by the publisher.
