# Supplementary material for: Cardiovascular toxicity associated with angiogenesis inhibitors: A comprehensive pharmacovigilance analysis based on the FDA Adverse Event Reporting System database from 2014 to 2021
Source: Front Cardiovasc Med. 2022 Oct 13;9:988013. doi: 10.3389/fcvm.2022.988013 (PMC9606330; doi:10.3389/fcvm.2022.988013)
Supplement: Supplementary file 2 [file Data_Sheet_2.PDF]

TABLE S10 Thorough drug name archive of angiogenesis inhibitors

Generic names	Brand names	Research codes
Bevacizumab	Avastin	
Bevacizumab-Awwb	Mvasi	
Bevacizumab-Bvzr	Zirabev	PF-06439535
Bevacizumab	Avastin	
	BYVASDA	
Bevacizumab (China)	Pusintin	
	POBEVCY	
Ramucirumab	Cyramza	IMC-1121B
Aflibercept	Zaltrap	BAY-865321
Sorafenib	Nexavar	BAY 43-9006
Sunitinib	Sutent	SU11248
Pazopanib	Votrient	GW786034
Vandetanib	Caprelsa	ZD6474
Cabozantinib	Cabometyx	XL184
Regorafenib	Stivarga	BAY 73-4506
Axitinib	Inlyta	AG-013736
Nintedanib	Vargatef/Ofev	BIBF 1120
Lenvatinib	Lenvima/Lenvanix	E7080
Cediranib	Recentin	AZD2171; NSC-732208
Tivozanib	Fotivda	AV-951; KRN-951
Erdafitinib	Balversa	JNJ-42756493
Vatalanib		PTK787; ZK-222584; CGP-79787
Apatinib (China)		YN968D1
Anlotinib (China)		AL3818
Fruquintinib (China)		HMPL-013
Recombinant human endostatin (China)	Endostar	YH-16
