# Supplementary material for: Cardiovascular toxicity associated with angiogenesis inhibitors: A comprehensive pharmacovigilance analysis based on the FDA Adverse Event Reporting System database from 2014 to 2021
Source: Front Cardiovasc Med. 2022 Oct 13;9:988013. doi: 10.3389/fcvm.2022.988013 (PMC9606330; doi:10.3389/fcvm.2022.988013)
Supplement: Supplementary file 3 [file Data_Sheet_3.PDF]

Drug	PT	N	IC	IC025	IC975	ROR	ROR025	ROR975
All agents (112)	Hypertension	10654	2.10	2.06	2.12	4.27	4.19	4.36
	Dyspnoea	7739	0.12	0.09	0.15	1.09	1.07	1.11
	Blood pressure increased	6404	1.78	1.74	1.81	3.44	3.35	3.53
	Pulmonary embolism	2250	1.01	0.94	1.06	2.01	1.93	2.10
	Ascites	2072	2.55	2.48	2.60	5.86	5.59	6.14
	Oedema peripheral	1745	0.58	0.50	0.64	1.50	1.43	1.57
	Cardiac failure	1308	0.44	0.35	0.51	1.36	1.29	1.44
	Thrombosis	1245	0.32	0.23	0.39	1.25	1.18	1.32
	Deep vein thrombosis	1133	0.50	0.40	0.57	1.41	1.33	1.50
	Dyspnoea exertional	938	1.02	0.91	1.10	2.03	1.90	2.17
	Oedema	848	0.42	0.31	0.50	1.34	1.25	1.43
	Pulmonary oedema	596	0.16	0.02	0.26	1.12	1.03	1.21
	Blood pressure fluctuation	561	0.73	0.59	0.83	1.65	1.52	1.80
	Cerebral infarction	544	0.84	0.70	0.94	1.79	1.64	1.95
	Transient ischaemic attack	534	0.53	0.38	0.63	1.44	1.32	1.57
	Disseminated intravascular coagulation	530	1.86	1.71	1.96	3.62	3.31	3.96
	Hypoxia	525	0.39	0.25	0.49	1.31	1.20	1.43
	Ejection fraction decreased	502	1.50	1.35	1.61	2.83	2.59	3.10
	Embolism	491	2.29	2.14	2.39	4.88	4.44	5.36
	Pericardial effusion	471	0.83	0.68	0.94	1.78	1.62	1.95
	Hypertensive crisis	440	1.61	1.45	1.72	3.04	2.76	3.35
	Blood pressure abnormal	408	0.72	0.56	0.84	1.65	1.49	1.82
	Mental status changes	388	0.35	0.18	0.47	1.28	1.15	1.41
	Hemiparesis	348	0.71	0.53	0.84	1.64	1.47	1.82
	Blood pressure systolic increased	339	0.59	0.41	0.72	1.50	1.35	1.68
	Thrombotic microangiopathy	301	1.41	1.22	1.55	2.66	2.37	2.99
	Cardiomyopathy	271	0.71	0.51	0.85	1.63	1.45	1.84
	Cardiotoxicity	243	1.32	1.11	1.48	2.50	2.20	2.85
	Acute coronary syndrome	231	1.14	0.92	1.30	2.20	1.93	2.52
	Sudden death	229	1.04	0.82	1.19	2.05	1.80	2.34
	Portal vein thrombosis	210	2.46	2.23	2.62	5.49	4.75	6.35
	Left ventricular dysfunction	208	1.34	1.11	1.50	2.53	2.20	2.91
	Venous thrombosis	178	1.82	1.58	2.00	3.54	3.04	4.13
	Pulmonary thrombosis	173	0.31	0.06	0.50	1.24	1.07	1.45
	Myocardial ischaemia	158	0.34	0.08	0.53	1.26	1.08	1.48
	Embolism venous	155	1.84	1.58	2.03	3.59	3.04	4.23
	Blood pressure diastolic increased	154	1.61	1.34	1.80	3.05	2.59	3.60
	Myocarditis	151	0.39	0.12	0.58	1.31	1.11	1.54
	Cerebral ischaemia	149	1.40	1.13	1.60	2.65	2.24	3.12
	Hemiplegia	147	0.53	0.26	0.73	1.45	1.23	1.70
	Cardiac failure acute	140	0.84	0.56	1.05	1.80	1.52	2.13

	Cardiopulmonary failure	125	1.42	1.12	1.63	2.67	2.23	3.20
	Venoocclusive liver disease	119	1.06	0.76	1.28	2.09	1.74	2.51
	Troponin increased	112	0.58	0.27	0.81	1.50	1.24	1.81
	Congestive cardiomyopathy	99	0.79	0.46	1.03	1.73	1.41	2.11
	Venous thrombosis limb	95	1.81	1.47	2.05	3.50	2.84	4.32
	Cardiac dysfunction	93	1.27	0.93	1.52	2.42	1.96	2.98
	Vena cava thrombosis	81	2.02	1.65	2.28	4.04	3.22	5.08
	Thrombotic thrombocytopenic purpura	79	0.96	0.59	1.23	1.95	1.55	2.44
	Arteriospasm coronary	78	0.98	0.60	1.25	1.97	1.57	2.47
	Retinal vein occlusion	75	1.33	0.95	1.61	2.52	1.99	3.18
	Coronary artery stenosis	73	0.74	0.35	1.02	1.67	1.32	2.11
	Superior vena cava syndrome	66	2.76	2.35	3.05	6.77	5.20	8.81
	Ventricular hypokinesia	62	0.88	0.46	1.18	1.84	1.42	2.37
	Embolism arterial	60	1.93	1.50	2.24	3.81	2.92	4.96
	Left ventricular failure	59	0.70	0.27	1.01	1.62	1.25	2.10
	Arterial thrombosis	57	1.29	0.85	1.60	2.44	1.87	3.19
	Jugular vein thrombosis	56	1.46	1.02	1.78	2.75	2.10	3.61
	Orthopnoea	55	0.52	0.07	0.84	1.43	1.09	1.87
	Retinal artery occlusion	49	0.83	0.35	1.17	1.77	1.33	2.36
	Cardiac ventricular thrombosis	46	1.86	1.37	2.21	3.64	2.69	4.92
	Mesenteric vein thrombosis	42	1.82	1.30	2.18	3.53	2.57	4.84
	Hypertensive encephalopathy	40	2.64	2.11	3.01	6.22	4.44	8.72
	Malignant hypertension	39	1.85	1.32	2.23	3.62	2.60	5.02
	Tumour thrombosis	38	3.61	3.07	4.00	12.24	8.30	18.05
	Pulmonary artery thrombosis	38	2.05	1.51	2.43	4.13	2.96	5.78
	Lacunar infarction	37	0.62	0.07	1.01	1.54	1.11	2.13
	Arrhythmia supraventricular	36	1.68	1.13	2.08	3.21	2.28	4.50
	Computerised tomogram thorax abnormal	36	1.52	0.96	1.91	2.86	2.04	4.02
	Subclavian vein thrombosis	34	1.23	0.65	1.63	2.34	1.66	3.31
	Tumour embolism	33	3.50	2.92	3.91	11.31	7.50	17.04
	Pelvic venous thrombosis	33	0.78	0.20	1.19	1.71	1.21	2.43
	Lung opacity	33	0.74	0.16	1.16	1.68	1.18	2.37
	Aortic thrombosis	30	1.28	0.67	1.71	2.43	1.68	3.51
	Ventricular dysfunction	30	0.85	0.24	1.28	1.80	1.25	2.60
	Device related thrombosis	29	1.19	0.57	1.62	2.27	1.56	3.31
	Secondary hypertension	28	2.77	2.14	3.21	6.81	4.53	10.25
	Hypertensive emergency	27	1.04	0.40	1.50	2.06	1.40	3.03
	Troponin T increased	26	0.93	0.27	1.39	1.90	1.28	2.82
	Thrombophlebitis migrans	23	3.03	2.33	3.52	8.19	5.15	13.02
	Labile blood pressure	23	0.79	0.09	1.28	1.73	1.14	2.62
	Splenic vein thrombosis	21	1.70	0.97	2.22	3.26	2.09	5.09
	Blood creatine phosphokinase MB increased	21	1.01	0.28	1.52	2.01	1.30	3.12
	Electrocardiogram change	20	1.21	0.46	1.74	2.32	1.47	3.63

	Hypertensive urgency	19	2.10	1.33	2.64	4.29	2.66	6.90
	Systolic hypertension	18	1.71	0.92	2.26	3.27	2.02	5.30
	Renal vein thrombosis	18	1.32	0.52	1.87	2.49	1.55	4.01
	Visual acuity reduced transiently	16	1.70	0.86	2.28	3.25	1.95	5.41
	Mesenteric artery thrombosis	16	1.66	0.82	2.24	3.16	1.90	5.27
	Retinal vein thrombosis	15	1.18	0.31	1.78	2.26	1.34	3.81
	Axillary vein thrombosis	13	1.42	0.48	2.07	2.68	1.53	4.71
	Brachiocephalic vein thrombosis	11	2.51	1.49	3.20	5.70	2.98	10.88
	Hepatic infarction	11	1.27	0.25	1.97	2.42	1.31	4.45
	Immune-mediated myocarditis	11	1.15	0.12	1.84	2.21	1.21	4.06
	Renal artery occlusion	10	1.73	0.65	2.45	3.31	1.73	6.34
	Rhythm idioventricular	10	1.38	0.31	2.11	2.61	1.38	4.96
	Accelerated hypertension	10	1.10	0.02	1.83	2.14	1.13	4.05
	Portal vein occlusion	9	2.17	1.03	2.93	4.50	2.23	9.06
	Autoimmune myocarditis	9	1.44	0.30	2.21	2.72	1.38	5.36
	Retinopathy hypertensive	9	1.38	0.24	2.14	2.60	1.32	5.11
	Pulmonary tumour thrombotic microangiopathy	8	2.68	1.47	3.48	6.40	2.92	14.04
	Collateral circulation	8	1.80	0.59	2.61	3.49	1.69	7.24
	Catheter site thrombosis	8	1.36	0.15	2.16	2.57	1.25	5.26
	Splenic artery thrombosis	7	2.47	1.17	3.32	5.55	2.44	12.63
	Vena cava embolism	7	2.42	1.12	3.28	5.37	2.37	12.16
	Myocardial injury	6	2.34	0.93	3.25	5.07	2.10	12.24
	Diastolic hypertension	6	1.75	0.34	2.67	3.37	1.45	7.83
	Choroidal infarction	6	1.50	0.09	2.41	2.83	1.23	6.51
	ECG signs of myocardial infarction	5	2.70	1.14	3.69	6.51	2.22	19.04
	Central venous pressure increased	5	1.91	0.34	2.89	3.75	1.47	9.54
	Cardiac ventricular scarring	4	2.58	0.81	3.66	5.97	1.75	20.41
	Paget-Schroetter syndrome	3	2.22	0.15	3.42	4.65	1.23	17.52
Bevacizumab (106)	Hypertension	3546	2.21	2.15	2.25	4.62	4.46	4.77
	Pulmonary embolism	1037	1.59	1.49	1.66	3.01	2.83	3.20
	Blood pressure increased	1024	0.84	0.73	0.91	1.78	1.68	1.90
	Ascites	738	2.76	2.63	2.84	6.76	6.27	7.28
	Deep vein thrombosis	679	1.46	1.33	1.55	2.74	2.54	2.96
	Oedema peripheral	565	0.65	0.52	0.76	1.57	1.45	1.71
	Thrombosis	498	0.70	0.55	0.80	1.62	1.48	1.77
	Cardiac failure	445	0.59	0.43	0.70	1.50	1.37	1.65
	Tachycardia	366	0.22	0.05	0.35	1.17	1.05	1.29
	Embolism	333	3.41	3.23	3.54	10.63	9.50	11.88
	Oedema	298	0.61	0.42	0.75	1.52	1.36	1.71
	Dyspnoea exertional	238	0.74	0.53	0.90	1.67	1.47	1.90
	Hemiparesis	219	1.73	1.51	1.90	3.33	2.91	3.81
	Hypoxia	217	0.81	0.59	0.97	1.76	1.54	2.01

	Disseminated intravascular coagulation	209	2.20	1.97	2.37	4.60	4.01	5.28
	Cerebral infarction	202	1.10	0.87	1.27	2.15	1.87	2.47
	Thrombotic microangiopathy	188	2.42	2.18	2.59	5.34	4.62	6.18
	Transient ischaemic attack	183	0.68	0.43	0.85	1.60	1.38	1.85
	Hypertensive crisis	182	2.02	1.78	2.20	4.06	3.51	4.71
	Mental status changes	179	0.93	0.68	1.11	1.91	1.64	2.21
	Ejection fraction decreased	165	1.59	1.33	1.78	3.01	2.58	3.51
	Pericardial effusion	149	0.86	0.59	1.06	1.82	1.55	2.14
	Venous thrombosis	125	2.98	2.69	3.20	7.90	6.60	9.47
	Embolism venous	122	3.16	2.86	3.38	8.94	7.44	10.74
	Ischaemic stroke	116	0.59	0.28	0.81	1.50	1.25	1.80
	Cardiomyopathy	110	1.10	0.78	1.33	2.14	1.78	2.59
	Cardiotoxicity	109	1.85	1.54	2.08	3.61	2.99	4.37
	Venoocclusive liver disease	108	2.59	2.27	2.82	6.03	4.97	7.31
	Acute coronary syndrome	106	1.70	1.38	1.93	3.26	2.68	3.95
	Cerebral ischaemia	99	2.49	2.15	2.73	5.60	4.58	6.85
	Left ventricular dysfunction	96	1.91	1.57	2.15	3.75	3.06	4.59
	Blood pressure systolic increased	93	0.42	0.08	0.67	1.34	1.09	1.64
	Ventricular fibrillation	91	1.34	0.99	1.59	2.53	2.05	3.11
	Sudden death	82	1.24	0.88	1.51	2.37	1.90	2.95
	Myocardial ischaemia	79	1.03	0.66	1.30	2.04	1.63	2.55
	Venous thrombosis limb	78	3.17	2.79	3.44	8.97	7.13	11.29
	Sinus tachycardia	78	0.74	0.36	1.01	1.67	1.34	2.09
	Hemiplegia	77	1.29	0.91	1.56	2.44	1.95	3.06
	Portal vein thrombosis	74	2.62	2.23	2.89	6.13	4.85	7.74
	Blood pressure diastolic increased	62	1.97	1.55	2.27	3.92	3.05	5.05
	Myocarditis	61	0.77	0.34	1.07	1.70	1.32	2.19
	Pulmonary thrombosis	60	0.48	0.05	0.79	1.40	1.08	1.80
	Arteriospasm coronary	59	2.23	1.80	2.54	4.70	3.63	6.10
	Blindness transient	55	1.02	0.57	1.34	2.03	1.56	2.65
	Congestive cardiomyopathy	51	1.51	1.04	1.84	2.85	2.16	3.75
	Atrial flutter	48	0.66	0.18	1.00	1.58	1.19	2.10
	Cardiac failure acute	47	0.96	0.47	1.30	1.94	1.46	2.59
	Stress cardiomyopathy	46	1.10	0.61	1.45	2.15	1.61	2.88
	Retinal vein occlusion	42	2.15	1.64	2.52	4.43	3.26	6.03
	Thrombotic thrombocytopenic purpura	41	1.68	1.16	2.05	3.21	2.35	4.37
	Thrombophlebitis	41	1.64	1.12	2.01	3.11	2.28	4.24
	Embolism arterial	37	2.84	2.29	3.23	7.15	5.13	9.96
	Superior vena cava syndrome	36	3.44	2.88	3.83	10.83	7.69	15.24
	Arterial thrombosis	34	2.18	1.61	2.59	4.53	3.22	6.38
	Coronary artery stenosis	34	1.31	0.74	1.72	2.48	1.77	3.48
	Arrhythmia supraventricular	33	3.12	2.54	3.53	8.69	6.10	12.38
	Vena cava thrombosis	30	2.22	1.61	2.65	4.65	3.23	6.70

	Jugular vein thrombosis	29	2.15	1.53	2.58	4.42	3.05	6.41
	Cardiac dysfunction	28	1.22	0.58	1.66	2.32	1.60	3.37
	Peripheral arterial occlusive disease	27	0.67	0.02	1.12	1.59	1.09	2.32
	Device related thrombosis	26	2.61	1.95	3.07	6.10	4.11	9.05
	Thrombosis in device	26	0.92	0.26	1.38	1.89	1.28	2.78
	Pulmonary artery thrombosis	25	2.99	2.32	3.46	7.93	5.29	11.89
	Left ventricular failure	23	1.01	0.31	1.50	2.02	1.34	3.05
	Cerebrovascular disorder	22	1.33	0.61	1.83	2.51	1.64	3.82
	Blood pressure diastolic abnormal	22	1.21	0.49	1.71	2.31	1.51	3.52
	Pelvic venous thrombosis	21	1.75	1.02	2.27	3.37	2.19	5.20
	Lung opacity	20	1.66	0.91	2.18	3.15	2.02	4.91
	Retinal artery occlusion	20	1.20	0.44	1.72	2.29	1.47	3.56
	Cardiac ventricular thrombosis	19	2.19	1.42	2.72	4.55	2.88	7.19
	Lacunar infarction	19	1.31	0.54	1.85	2.48	1.57	3.90
	Thrombophlebitis migrans	18	3.91	3.12	4.46	15.04	9.04	25.00
	Hypertensive encephalopathy	18	2.98	2.18	3.53	7.87	4.88	12.70
	Subclavian vein thrombosis	18	1.92	1.13	2.47	3.78	2.37	6.05
	Malignant hypertension	17	2.24	1.42	2.80	4.71	2.90	7.65
	Monoparesis	17	1.30	0.48	1.87	2.46	1.52	3.97
	Mesenteric vein thrombosis	16	2.02	1.18	2.61	4.06	2.47	6.69
	Splenic infarction	16	1.23	0.39	1.81	2.34	1.43	3.84
	Electrocardiogram ST segment depression	16	1.14	0.30	1.73	2.21	1.35	3.62
	Pulmonary infarction	15	0.88	0.01	1.48	1.84	1.11	3.07
	Aortic thrombosis	14	1.78	0.88	2.40	3.44	2.02	5.85
	Computerised tomogram thorax abnormal	14	1.76	0.86	2.38	3.39	2.00	5.77
	Troponin I increased	14	1.29	0.38	1.91	2.44	1.44	4.14
	Visual acuity reduced transiently	13	2.82	1.88	3.46	7.06	4.03	12.40
	Ventricular hypertrophy	13	1.66	0.72	2.30	3.16	1.82	5.48
	Axillary vein thrombosis	11	2.60	1.58	3.29	6.06	3.30	11.14
	Pulmonary valve incompetence	11	1.43	0.41	2.13	2.70	1.48	4.90
	Labile blood pressure	11	1.34	0.32	2.04	2.53	1.39	4.60
	Ventricular dysfunction	11	1.05	0.02	1.74	2.07	1.14	3.75
	Troponin T increased	10	1.18	0.10	1.90	2.26	1.21	4.23
	Brachiocephalic vein thrombosis	9	3.29	2.16	4.06	9.81	4.86	19.82
	Secondary hypertension	9	2.54	1.40	3.30	5.81	2.97	11.39
	Right ventricular dilatation	9	1.59	0.45	2.35	3.01	1.55	5.83
	Intestinal infarction	8	1.29	0.08	2.10	2.45	1.22	4.93
	Splenic artery thrombosis	7	3.30	2.00	4.15	9.84	4.32	22.40
	Catheter site thrombosis	7	2.46	1.16	3.31	5.49	2.56	11.78
	Retinopathy hypertensive	7	2.35	1.05	3.20	5.10	2.38	10.90
	Choroidal infarction	6	2.63	1.21	3.54	6.17	2.68	14.20
	Collateral circulation	6	2.57	1.15	3.48	5.93	2.59	13.61
	Tumour thrombosis	6	2.30	0.89	3.22	4.94	2.17	11.24

	Hypertensive urgency	6	1.89	0.48	2.81	3.72	1.65	8.39
	Pulmonary tumour thrombotic microangiopathy	5	2.86	1.30	3.85	7.28	2.83	18.73
	Tumour embolism	5	2.13	0.57	3.11	4.37	1.78	10.72
	Hepatic infarction	5	1.60	0.04	2.59	3.04	1.25	7.38
	Renal artery occlusion	4	1.77	0.00	2.85	3.40	1.26	9.22
	Vena cava embolism	3	2.17	0.10	3.38	4.51	1.39	14.64
Ramucirumab (25)	Hypertension	144	1.97	1.69	2.17	3.92	3.32	4.62
	Ascites	113	4.33	4.02	4.55	20.08	16.67	24.18
	Pulmonary embolism	63	1.92	1.50	2.22	3.77	2.95	4.84
	Disseminated intravascular coagulation	51	4.29	3.83	4.62	19.57	14.84	25.79
	Cardiac failure	51	1.82	1.36	2.16	3.54	2.69	4.66
	Oedema	46	2.25	1.76	2.60	4.77	3.57	6.37
	Oedema peripheral	38	1.14	0.60	1.52	2.20	1.60	3.03
	Deep vein thrombosis	36	1.58	1.03	1.98	2.99	2.16	4.15
	Thrombosis	25	0.76	0.09	1.23	1.70	1.14	2.51
	Thrombotic microangiopathy	21	3.32	2.59	3.84	10.01	6.52	15.38
	Transient ischaemic attack	16	1.48	0.64	2.06	2.79	1.71	4.56
	Cerebral infarction	15	1.65	0.78	2.25	3.14	1.89	5.21
	Hypoxia	14	1.19	0.29	1.81	2.29	1.35	3.86
	Sudden death	11	2.44	1.41	3.13	5.41	2.99	9.78
	Embolism venous	10	3.22	2.14	3.95	9.33	5.01	17.38
	Portal vein thrombosis	9	3.17	2.04	3.94	9.03	4.69	17.39
	Embolism	7	1.93	0.63	2.79	3.82	1.82	8.02
	Jugular vein thrombosis	6	3.04	1.62	3.95	8.21	3.68	18.32
	Thrombotic thrombocytopenic purpura	6	2.58	1.16	3.49	5.97	2.68	13.31
	Cardiac failure acute	6	1.99	0.58	2.90	3.98	1.78	8.86
	Nocturnal dyspnoea	5	2.90	1.34	3.88	7.45	3.09	17.97
	Venous thrombosis	5	2.16	0.60	3.15	4.48	1.86	10.77
	Left ventricular dysfunction	5	1.70	0.14	2.68	3.24	1.35	7.80
	Vena cava thrombosis	4	2.52	0.75	3.59	5.72	2.14	15.28
	Pulmonary artery thrombosis	3	2.48	0.41	3.68	5.57	1.79	17.34
Aflibercept (47)	Hypertension	309	2.85	2.66	2.99	7.22	6.45	8.09
	Pulmonary embolism	105	2.44	2.11	2.67	5.41	4.46	6.55
	Cardiac failure	45	1.43	0.94	1.79	2.70	2.02	3.62
	Deep vein thrombosis	41	1.55	1.03	1.93	2.94	2.16	3.99
	Hypertensive crisis	36	3.63	3.07	4.02	12.37	8.91	17.18
	Atrial fibrillation	36	0.85	0.29	1.24	1.80	1.30	2.49
	Oedema peripheral	34	0.77	0.19	1.17	1.70	1.21	2.38
	Tachycardia	33	0.91	0.33	1.33	1.88	1.34	2.65
	Transient ischaemic attack	30	2.16	1.56	2.60	4.48	3.13	6.42
	Cerebral infarction	25	2.17	1.50	2.64	4.50	3.04	6.66

	Blindness transient	24	3.63	2.95	4.11	12.37	8.28	18.49
	Ascites	23	1.85	1.16	2.34	3.61	2.40	5.44
	Blood creatine phosphokinase increased	22	2.10	1.39	2.60	4.30	2.83	6.53
	Ejection fraction decreased	20	2.55	1.80	3.07	5.85	3.77	9.07
	Acute coronary syndrome	19	3.10	2.33	3.64	8.57	5.46	13.45
	Vena cava thrombosis	16	4.31	3.46	4.89	19.78	12.06	32.46
	Cardiac ventricular thrombosis	13	4.25	3.31	4.89	19.05	10.98	33.03
	Angina pectoris	13	1.14	0.20	1.78	2.20	1.28	3.80
	Ischaemic stroke	12	1.40	0.42	2.06	2.63	1.49	4.63
	Retinal artery occlusion	11	3.57	2.55	4.27	11.90	6.57	21.56
	Retinal vein occlusion	11	3.52	2.50	4.22	11.49	6.34	20.80
	Sudden death	11	2.26	1.24	2.96	4.81	2.66	8.69
	Haemorrhagic stroke	11	2.24	1.21	2.93	4.71	2.61	8.52
	Orthostatic hypotension	11	1.55	0.53	2.25	2.94	1.62	5.30
	Arterial occlusive disease	10	2.35	1.28	3.08	5.11	2.75	9.51
	Embolism	10	2.25	1.18	2.98	4.77	2.56	8.86
	Superior vena cava syndrome	8	3.69	2.48	4.49	12.91	6.41	26.00
	Troponin increased	8	2.27	1.05	3.07	4.81	2.40	9.63
	Left ventricular dysfunction	8	2.17	0.95	2.97	4.49	2.24	8.99
	Embolism arterial	7	3.30	2.00	4.15	9.85	4.68	20.74
	Jugular vein thrombosis	7	3.16	1.86	4.01	8.93	4.24	18.78
	Subclavian vein thrombosis	6	3.13	1.72	4.04	8.76	3.92	19.58
	Atrial thrombosis	6	2.64	1.22	3.55	6.22	2.79	13.87
	Aortic thrombosis	5	2.96	1.40	3.95	7.80	3.23	18.83
	Thrombosis in device	5	2.14	0.58	3.13	4.42	1.84	10.62
	Cardiac failure chronic	5	1.95	0.39	2.94	3.87	1.61	9.30
	Congestive cardiomyopathy	5	1.90	0.34	2.89	3.74	1.55	8.99
	Myocardial necrosis	4	3.00	1.23	4.08	7.99	2.96	21.57
	Mesenteric artery thrombosis	4	2.96	1.19	4.03	7.76	2.88	20.87
	Left ventricular dilatation	4	2.83	1.07	3.91	7.12	2.66	19.08
	Ventricular dysfunction	4	2.53	0.76	3.61	5.77	2.16	15.43
	Portal vein thrombosis	4	1.98	0.21	3.06	3.94	1.48	10.50
	Splenic thrombosis	3	2.71	0.64	3.91	6.53	2.07	20.62
	Visual acuity reduced transiently	3	2.60	0.53	3.81	6.06	1.94	18.95
	Malignant hypertension	3	2.37	0.30	3.58	5.17	1.66	16.09
	Cardiac aneurysm	3	2.34	0.27	3.55	5.07	1.63	15.77
	Left atrial dilatation	3	2.24	0.17	3.45	4.73	1.52	14.71
Sunitinib (27)	Hypertension	1340	1.79	1.70	1.85	3.46	3.27	3.65
	Blood pressure increased	1039	1.84	1.74	1.92	3.59	3.37	3.81
	Peripheral swelling	464	0.39	0.24	0.50	1.31	1.20	1.44
	Oedema peripheral	292	0.69	0.50	0.83	1.61	1.44	1.81
	Ascites	245	2.15	1.94	2.30	4.43	3.91	5.03

	Pulmonary oedema	170	1.03	0.78	1.21	2.04	1.76	2.38
	Oedema	160	0.70	0.43	0.89	1.62	1.39	1.89
	Pericardial effusion	111	1.42	1.11	1.65	2.68	2.22	3.23
	Cardiopulmonary failure	94	3.61	3.27	3.86	12.22	9.94	15.03
	Blood pressure fluctuation	83	0.65	0.29	0.91	1.57	1.27	1.95
	Blood pressure systolic increased	79	1.16	0.79	1.43	2.24	1.79	2.80
	Disseminated intravascular coagulation	77	1.74	1.36	2.01	3.34	2.67	4.18
	Blood pressure abnormal	69	0.83	0.44	1.12	1.78	1.41	2.26
	Ejection fraction decreased	65	1.23	0.81	1.52	2.34	1.83	2.99
	Hypertensive crisis	51	1.17	0.70	1.50	2.25	1.71	2.96
	Pulmonary thrombosis	41	0.91	0.39	1.28	1.88	1.38	2.55
	Thrombotic microangiopathy	32	0.85	0.26	1.27	1.80	1.27	2.55
	Sudden death	28	0.68	0.05	1.12	1.60	1.10	2.32
	Blood pressure diastolic decreased	25	1.02	0.35	1.49	2.02	1.36	3.00
	Cardiotoxicity	25	0.72	0.05	1.19	1.64	1.11	2.43
	Monoplegia	16	0.96	0.12	1.54	1.95	1.19	3.18
	Thrombotic thrombocytopenic purpura	15	1.19	0.32	1.80	2.29	1.38	3.80
	Vena cava thrombosis	14	2.03	1.13	2.65	4.08	2.41	6.92
	Malignant hypertension	6	1.62	0.20	2.53	3.07	1.37	6.86
	Renal vein thrombosis	5	1.80	0.24	2.79	3.49	1.44	8.44
	Tumour embolism	4	2.35	0.58	3.43	5.10	1.88	13.84
	Mesenteric artery stenosis	3	2.35	0.28	3.56	5.10	1.59	16.37
Lenvatinib (35)	Hypertension	1585	3.09	3.01	3.15	8.54	8.12	8.98
	Blood pressure increased	1418	3.35	3.27	3.42	10.22	9.69	10.78
	Peripheral swelling	224	0.40	0.18	0.56	1.32	1.16	1.51
	Ascites	198	2.89	2.66	3.06	7.42	6.45	8.54
	Pulmonary embolism	198	1.25	1.01	1.42	2.37	2.06	2.73
	Chest pain	194	0.29	0.06	0.46	1.22	1.06	1.41
	Cerebrovascular accident	192	0.39	0.15	0.56	1.31	1.14	1.51
	Blood pressure fluctuation	180	2.81	2.57	2.99	7.03	6.06	8.14
	Oedema peripheral	161	0.89	0.63	1.08	1.86	1.59	2.17
	Cardiac failure	128	0.84	0.54	1.05	1.79	1.50	2.12
	Oedema	77	0.70	0.32	0.97	1.63	1.30	2.04
	Blood pressure abnormal	73	1.96	1.57	2.24	3.89	3.09	4.90
	Cerebral infarction	66	1.53	1.12	1.82	2.88	2.26	3.67
	Acute myocardial infarction	51	0.91	0.44	1.24	1.87	1.42	2.47
	Transient ischaemic attack	51	0.88	0.41	1.21	1.84	1.39	2.42
	Hypertensive crisis	41	1.89	1.37	2.26	3.70	2.72	5.03
	Portal vein thrombosis	39	3.58	3.04	3.96	11.93	8.68	16.39
	Blood pressure diastolic increased	39	3.23	2.69	3.61	9.36	6.82	12.84
	Mental status changes	35	0.62	0.06	1.02	1.54	1.10	2.14
	Blood pressure systolic increased	32	0.92	0.33	1.33	1.89	1.33	2.67

	Ejection fraction decreased	28	1.06	0.43	1.51	2.09	1.44	3.03
	Disseminated intravascular coagulation	22	0.99	0.27	1.49	1.98	1.30	3.01
	Embolism	19	1.29	0.52	1.83	2.45	1.56	3.85
	Acute coronary syndrome	18	1.16	0.37	1.71	2.24	1.41	3.55
	Sudden death	18	1.08	0.28	1.63	2.11	1.33	3.35
	Cardiac dysfunction	16	2.30	1.46	2.89	4.94	3.02	8.09
	Haemorrhagic stroke	16	0.88	0.03	1.46	1.84	1.12	3.00
	Sinus arrest	11	2.72	1.69	3.41	6.58	3.63	11.94
	Rhythm idioventricular	10	3.79	2.71	4.51	13.79	7.26	26.17
	Hypertensive emergency	6	2.16	0.75	3.07	4.47	2.00	9.99
	Hypertensive urgency	5	2.78	1.22	3.76	6.87	2.82	16.71
	Aortic thrombosis	5	1.98	0.42	2.96	3.94	1.63	9.50
	Portal vein occlusion	4	2.86	1.10	3.94	7.26	2.65	19.92
	Hypertensive encephalopathy	4	2.25	0.48	3.33	4.75	1.77	12.77
	Portopulmonary hypertension	3	2.52	0.45	3.72	5.73	1.80	18.24
Nintedanib (38)	Dyspnoea	1854	1.56	1.48	1.61	2.94	2.81	3.08
	Dyspnoea exertional	408	3.30	3.14	3.42	9.85	8.93	10.87
	Chest pain	346	0.87	0.69	1.00	1.83	1.64	2.03
	Hypertension	312	0.50	0.31	0.63	1.41	1.26	1.58
	Blood pressure increased	234	0.50	0.29	0.66	1.42	1.24	1.61
	Cerebrovascular accident	216	0.31	0.08	0.47	1.24	1.08	1.41
	Pulmonary embolism	190	0.93	0.69	1.11	1.91	1.66	2.20
	Atrial fibrillation	141	0.45	0.17	0.65	1.36	1.15	1.61
	Hypoxia	120	1.74	1.44	1.96	3.34	2.79	4.00
	Pulmonary hypertension	116	2.30	1.99	2.52	4.91	4.09	5.90
	Cardiac failure	113	0.40	0.09	0.63	1.32	1.10	1.59
	Transient ischaemic attack	92	1.47	1.12	1.72	2.77	2.26	3.40
	Cerebral infarction	52	0.93	0.47	1.26	1.91	1.45	2.51
	Pulmonary arterial hypertension	39	0.90	0.36	1.28	1.86	1.36	2.55
	Blood pressure abnormal	39	0.81	0.28	1.19	1.76	1.28	2.41
	Pulmonary thrombosis	31	1.29	0.70	1.72	2.45	1.72	3.49
	Pulseless electrical activity	26	2.22	1.56	2.68	4.65	3.16	6.84
	Embolism	21	1.19	0.46	1.71	2.29	1.49	3.51
	Acute coronary syndrome	20	1.07	0.32	1.59	2.10	1.35	3.25
	Right ventricular failure	19	1.03	0.26	1.57	2.05	1.31	3.22
	Venous thrombosis	19	1.98	1.21	2.52	3.96	2.52	6.21
	Atrioventricular block	18	1.17	0.37	1.72	2.24	1.41	3.56
	Arteriosclerosis coronary artery	15	1.32	0.45	1.92	2.50	1.50	4.15
	Heart rate abnormal	14	1.13	0.23	1.75	2.19	1.30	3.70
	Mesenteric vein thrombosis	14	3.25	2.35	3.87	9.51	5.59	16.17
	Dyspnoea at rest	14	1.83	0.93	2.45	3.56	2.10	6.02
	Portal vein thrombosis	13	1.81	0.87	2.45	3.50	2.03	6.04

	Embolism venous	13	1.65	0.71	2.29	3.14	1.82	5.41
	Splenic vein thrombosis	10	3.33	2.26	4.06	10.08	5.36	18.97
	Paraparesis	9	1.95	0.81	2.71	3.85	2.00	7.42
	Thrombotic thrombocytopenic purpura	9	1.22	0.08	1.98	2.33	1.21	4.48
	Peripheral artery thrombosis	8	1.79	0.58	2.60	3.46	1.73	6.95
	Embolism arterial	6	1.80	0.39	2.71	3.48	1.56	7.78
	Dizziness exertional	6	2.39	0.97	3.30	5.23	2.33	11.71
	Cor pulmonale	5	1.86	0.29	2.84	3.62	1.50	8.74
	Thrombolysis	3	2.08	0.01	3.28	4.22	1.35	13.23
	Paroxysmal arrhythmia	3	2.54	0.47	3.75	5.83	1.81	18.75
	Myocardial injury	3	2.62	0.55	3.83	6.15	1.88	20.11
Pazopanib (39)	Hypertension	1103	2.27	2.17	2.35	4.83	4.55	5.13
	Dyspnoea	720	0.15	0.03	0.24	1.11	1.03	1.19
	Blood pressure increased	656	1.94	1.82	2.04	3.85	3.56	4.16
	Oedema peripheral	155	0.54	0.27	0.73	1.45	1.24	1.70
	Cardiac failure	154	0.81	0.54	1.00	1.75	1.49	2.05
	Ascites	127	1.96	1.67	2.17	3.89	3.27	4.63
	Ejection fraction decreased	76	2.20	1.82	2.47	4.59	3.66	5.75
	Disseminated intravascular coagulation	71	2.37	1.97	2.65	5.16	4.09	6.52
	Hypertensive crisis	66	2.28	1.87	2.58	4.86	3.82	6.20
	Transient ischaemic attack	53	0.64	0.18	0.96	1.56	1.19	2.04
	Pericardial effusion	52	1.09	0.63	1.42	2.13	1.62	2.79
	Blood pressure abnormal	48	1.07	0.59	1.41	2.10	1.58	2.79
	Cardiomyopathy	37	1.26	0.71	1.65	2.40	1.74	3.31
	Blood pressure systolic increased	36	0.79	0.24	1.19	1.73	1.25	2.40
	Left ventricular dysfunction	34	2.11	1.54	2.52	4.32	3.08	6.05
	Cardiotoxicity	27	1.56	0.92	2.01	2.95	2.02	4.30
	Troponin increased	26	1.86	1.20	2.32	3.63	2.46	5.33
	Ventricular hypokinesia	19	2.45	1.68	2.99	5.47	3.48	8.60
	Cardiac failure acute	19	1.36	0.59	1.90	2.56	1.63	4.03
	Cardiac dysfunction	16	2.05	1.21	2.63	4.13	2.53	6.76
	Orthopnoea	15	1.95	1.08	2.56	3.87	2.33	6.44
	Vena cava thrombosis	12	2.44	1.47	3.11	5.44	3.08	9.63
	Monoplegia	11	1.15	0.12	1.84	2.22	1.23	4.01
	Paresis	9	1.73	0.60	2.50	3.33	1.73	6.41
	Computerised tomogram thorax abnormal	8	2.39	1.18	3.20	5.25	2.61	10.56
	Electrocardiogram T wave inversion	8	1.70	0.49	2.50	3.24	1.62	6.51
	Brain natriuretic peptide increased	8	1.31	0.10	2.12	2.49	1.24	4.98
	Ventricular dysfunction	7	1.91	0.60	2.76	3.75	1.78	7.89
	Peripheral artery occlusion	7	1.63	0.33	2.49	3.10	1.47	6.53
	Hypertensive encephalopathy	6	2.63	1.22	3.54	6.20	2.76	13.92
	Renal infarct	6	1.61	0.19	2.52	3.05	1.36	6.80

	Malignant hypertension	5	1.92	0.36	2.91	3.79	1.57	9.14
	Cardiac ventricular thrombosis	5	1.76	0.20	2.75	3.39	1.40	8.18
	Left atrial dilatation	5	1.62	0.06	2.60	3.07	1.27	7.40
	Electrocardiogram change	4	1.83	0.07	2.91	3.56	1.33	9.54
	Cardiac ventricular scarring	3	2.74	0.67	3.95	6.69	1.77	25.22
	Ventricular dyskinesia	3	2.35	0.28	3.56	5.09	1.61	16.10
	Tumour thrombosis	3	2.24	0.17	3.44	4.72	1.50	14.85
	Renal artery occlusion	3	2.23	0.16	3.44	4.70	1.49	14.81
Cabozantinib (15)	Blood pressure increased	935	2.58	2.47	2.66	5.99	5.61	6.39
	Hypertension	701	1.75	1.62	1.84	3.36	3.11	3.62
	Pulmonary embolism	236	1.33	1.11	1.49	2.51	2.21	2.86
	Peripheral swelling	225	0.24	0.02	0.40	1.18	1.04	1.35
	Thrombosis	136	0.70	0.42	0.91	1.63	1.37	1.93
	Blood pressure fluctuation	88	1.62	1.26	1.87	3.07	2.49	3.78
	Ascites	61	1.03	0.61	1.34	2.05	1.59	2.63
	Blood pressure abnormal	54	1.36	0.91	1.69	2.57	1.97	3.36
	Embolism	41	2.22	1.70	2.59	4.67	3.43	6.35
	Ejection fraction decreased	29	0.95	0.33	1.39	1.93	1.34	2.78
	Sudden death	21	1.13	0.40	1.64	2.19	1.43	3.36
	Portal vein thrombosis	9	1.37	0.23	2.14	2.59	1.35	4.99
	Hypertensive urgency	4	2.42	0.66	3.50	5.36	1.99	14.46
	Mesenteric artery stenosis	3	2.54	0.47	3.75	5.83	1.82	18.71
	Pulmonary venous thrombosis	3	2.39	0.33	3.60	5.26	1.66	16.64
Sorafenib (37)	Hypertension	549	1.61	1.47	1.71	3.05	2.81	3.32
	Ascites	373	3.85	3.68	3.97	14.41	13.00	15.97
	Blood pressure increased	322	1.26	1.08	1.40	2.40	2.15	2.68
	Peripheral swelling	239	0.54	0.33	0.70	1.46	1.28	1.65
	Oedema peripheral	165	0.97	0.71	1.16	1.96	1.68	2.29
	Cerebral infarction	56	1.33	0.89	1.65	2.52	1.94	3.28
	Portal vein thrombosis	46	3.85	3.36	4.20	14.42	10.76	19.34
	Acute myocardial infarction	44	0.74	0.24	1.10	1.67	1.24	2.25
	Blood pressure fluctuation	38	0.63	0.09	1.01	1.55	1.12	2.13
	Blood pressure systolic increased	30	0.87	0.26	1.30	1.83	1.28	2.61
	Cardiotoxicity	30	2.03	1.42	2.46	4.09	2.85	5.85
	Hepatomegaly	29	1.84	1.22	2.28	3.58	2.48	5.15
	Thrombotic microangiopathy	27	1.67	1.03	2.13	3.19	2.18	4.65
	Ejection fraction decreased	25	0.95	0.28	1.42	1.93	1.30	2.85
	Tumour thrombosis	24	5.15	4.47	5.63	35.48	22.63	55.63
	Disseminated intravascular coagulation	22	1.03	0.32	1.53	2.04	1.34	3.10
	Cardiomyopathy	21	0.79	0.06	1.30	1.73	1.13	2.65
	Acute coronary syndrome	20	1.35	0.60	1.88	2.55	1.65	3.96

	Myocardial ischaemia	18	0.96	0.17	1.52	1.95	1.23	3.10
	Tumour embolism	15	4.52	3.65	5.12	22.89	13.26	39.50
	Cardiac failure acute	14	1.25	0.35	1.87	2.38	1.41	4.02
	Left ventricular dysfunction	13	1.08	0.14	1.72	2.11	1.22	3.64
	Blood creatine phosphokinase MB increased	12	3.34	2.36	4.01	10.13	5.71	17.98
	Systolic hypertension	8	3.29	2.07	4.09	9.76	4.82	19.76
	Retinal vein occlusion	8	1.70	0.48	2.50	3.24	1.62	6.50
	Ventricular hypokinesia	7	1.36	0.06	2.22	2.57	1.22	5.41
	Mesenteric vein thrombosis	7	2.49	1.19	3.35	5.63	2.67	11.88
	Retinal vein thrombosis	6	2.76	1.35	3.67	6.79	3.02	15.25
	Arterial thrombosis	6	1.59	0.17	2.50	3.01	1.35	6.71
	Electrocardiogram change	6	2.56	1.15	3.47	5.90	2.63	13.22
	Venous occlusion	6	1.55	0.14	2.46	2.93	1.31	6.54
	Cardiac ventricular thrombosis	5	1.99	0.43	2.97	3.97	1.65	9.58
	Systolic dysfunction	5	1.76	0.20	2.74	3.38	1.40	8.15
	Secondary hypertension	4	2.56	0.79	3.64	5.88	2.18	15.87
	Hepatic artery thrombosis	3	2.27	0.20	3.47	4.81	1.53	15.12
	Hepatic infarction	3	2.12	0.05	3.32	4.34	1.39	13.58
	Portal vein occlusion	3	2.51	0.44	3.71	5.68	1.79	18.07
Axitinib (24)	Hypertension	750	2.72	2.60	2.80	6.58	6.11	7.07
	Blood pressure increased	427	2.32	2.16	2.44	5.01	4.55	5.51
	Cerebrovascular accident	112	0.32	0.01	0.54	1.25	1.04	1.50
	Cardiac failure	84	0.93	0.57	1.19	1.90	1.54	2.36
	Pulmonary oedema	46	0.90	0.41	1.25	1.87	1.40	2.50
	Cerebral infarction	42	1.56	1.05	1.93	2.96	2.18	4.01
	Blood pressure abnormal	41	1.82	1.30	2.19	3.52	2.59	4.78
	Myocarditis	33	2.54	1.96	2.95	5.81	4.12	8.18
	Ascites	33	1.01	0.43	1.42	2.02	1.43	2.84
	Troponin increased	19	2.32	1.55	2.86	5.00	3.19	7.85
	Hypertensive crisis	18	1.39	0.59	1.94	2.62	1.65	4.15
	Ejection fraction decreased	17	1.03	0.21	1.60	2.04	1.27	3.29
	Sudden death	16	1.56	0.72	2.15	2.96	1.81	4.83
	Cardiac dysfunction	13	2.59	1.65	3.23	6.02	3.49	10.39
	Acute coronary syndrome	12	1.25	0.27	1.91	2.37	1.35	4.18
	Cardiac failure acute	10	1.38	0.30	2.10	2.60	1.40	4.83
	Blood pressure diastolic increased	10	1.92	0.84	2.64	3.77	2.03	7.02
	Essential hypertension	7	1.93	0.62	2.78	3.80	1.81	7.99
	Pulmonary infarction	5	1.72	0.16	2.70	3.29	1.37	7.92
	Hypertensive encephalopathy	4	2.54	0.77	3.62	5.81	2.17	15.61
	Autoimmune myocarditis	4	2.82	1.06	3.90	7.07	2.61	19.13
	Immune-mediated myocarditis	3	2.29	0.22	3.50	4.90	1.57	15.33
	Pericardial disease	3	2.43	0.36	3.64	5.40	1.72	16.96

	Tumour embolism	3	2.52	0.45	3.72	5.72	1.81	18.01
Regorafenib (23)	Hypertension	553	1.99	1.85	2.10	3.98	3.66	4.33
	Blood pressure increased	346	1.74	1.56	1.87	3.34	3.00	3.71
	Ascites	169	3.07	2.82	3.26	8.43	7.24	9.81
	Oedema peripheral	115	0.83	0.52	1.05	1.77	1.48	2.13
	Disseminated intravascular coagulation	62	2.86	2.44	3.16	7.26	5.65	9.32
	Dyspnoea exertional	58	1.16	0.73	1.48	2.24	1.73	2.90
	Cerebral infarction	34	0.99	0.42	1.39	1.98	1.42	2.78
	Blood pressure fluctuation	31	0.70	0.11	1.13	1.63	1.15	2.32
	Blood pressure systolic increased	29	1.18	0.56	1.62	2.27	1.58	3.27
	Ejection fraction decreased	20	0.99	0.24	1.51	1.98	1.28	3.08
	Hypertensive crisis	17	1.04	0.22	1.61	2.06	1.28	3.31
	Hepatomegaly	17	1.43	0.62	2.00	2.70	1.68	4.35
	Congestive cardiomyopathy	15	2.08	1.21	2.68	4.22	2.54	7.01
	Embolism	13	1.15	0.22	1.80	2.22	1.29	3.83
	Blood pressure diastolic increased	9	1.53	0.39	2.30	2.89	1.50	5.57
	Dyspnoea at rest	7	1.45	0.15	2.31	2.74	1.30	5.76
	Tumour embolism	6	3.35	1.94	4.26	10.21	4.48	23.26
	Ventricular hypokinesia	6	1.46	0.05	2.37	2.75	1.23	6.13
	Systolic hypertension	5	2.81	1.24	3.79	6.99	2.88	16.97
	Mesenteric vein thrombosis	4	1.98	0.21	3.06	3.94	1.47	10.54
	Hypertensive emergency	4	1.89	0.13	2.97	3.71	1.39	9.92
	Tumour thrombosis	4	2.80	1.03	3.88	6.96	2.56	18.86
	Angiotensin converting enzyme increased	3	2.57	0.50	3.77	5.92	1.86	18.83
Apatinib (domestic) (35)	Ejection fraction decreased	70	4.55	4.16	4.84	23.48	18.54	29.73
	Cardiotoxicity	35	4.25	3.69	4.65	19.02	13.63	26.54
	Pulmonary embolism	33	1.05	0.47	1.46	2.07	1.47	2.91
	Pericardial effusion	33	2.99	2.41	3.40	7.93	5.63	11.17
	Ascites	30	2.48	1.87	2.91	5.59	3.90	8.00
	Electrocardiogram QT prolonged	28	2.06	1.43	2.51	4.18	2.88	6.06
	Oedema peripheral	27	0.71	0.07	1.16	1.63	1.12	2.38
	Deep vein thrombosis	27	1.23	0.58	1.68	2.34	1.60	3.41
	Cardiac failure	23	0.75	0.05	1.24	1.68	1.12	2.53
	Cardiac arrest	22	0.73	0.01	1.23	1.66	1.09	2.52
	Oedema	21	1.19	0.46	1.71	2.29	1.49	3.51
	Left ventricular dysfunction	17	3.40	2.59	3.97	10.58	6.57	17.04
	Hypoxia	16	1.43	0.59	2.01	2.70	1.65	4.40
	Superior vena cava syndrome	13	4.41	3.47	5.05	21.27	12.24	36.95
	Cardiomyopathy	12	2.14	1.17	2.81	4.42	2.51	7.79
	Ejection fraction abnormal	11	3.99	2.97	4.69	15.91	8.77	28.87

	Pericarditis	9	1.96	0.82	2.72	3.89	2.02	7.49
	Left ventricular failure	9	3.23	2.09	4.00	9.41	4.88	18.12
	Tricuspid valve incompetence	8	2.52	1.31	3.33	5.75	2.87	11.52
	Arteriospasm coronary	8	3.01	1.80	3.81	8.06	4.02	16.14
	Secondary hypertension	7	3.76	2.46	4.61	13.54	6.35	28.87
	Computerised tomogram thorax abnormal	7	3.48	2.17	4.33	11.13	5.28	23.47
	Myocardial ischaemia	6	1.53	0.11	2.44	2.89	1.30	6.43
	Embolism	6	1.77	0.35	2.68	3.41	1.53	7.59
	Dyspnoea at rest	6	2.61	1.19	3.52	6.10	2.73	13.59
	Cardiomegaly	6	1.53	0.11	2.44	2.88	1.29	6.42
	Venous thrombosis	5	2.19	0.63	3.18	4.58	1.90	11.01
	Orthopnoea	5	2.40	0.84	3.39	5.30	2.20	12.74
	Diastolic dysfunction	5	2.50	0.94	3.49	5.66	2.35	13.63
	Cardiac tamponade	5	2.07	0.51	3.05	4.19	1.74	10.09
	Ventricular hypokinesia	4	2.21	0.45	3.29	4.63	1.74	12.36
	Venoocclusive liver disease	4	1.79	0.03	2.87	3.47	1.30	9.25
	Subclavian vein thrombosis	4	2.68	0.92	3.76	6.42	2.40	17.16
	Lacunar infarction	4	2.43	0.66	3.51	5.38	2.01	14.36
	Left ventricular enlargement	3	2.66	0.59	3.86	6.30	2.01	19.74
Vandetanib (9)	Electrocardiogram QT prolonged	87	4.78	4.42	5.04	27.47	22.21	33.98
	Hypertension	57	1.90	1.46	2.22	3.74	2.88	4.86
	Blood pressure increased	37	1.69	1.14	2.08	3.23	2.34	4.47
	Hemiparesis	20	3.54	2.79	4.06	11.60	7.47	18.01
	Deep vein thrombosis	15	1.55	0.68	2.15	2.93	1.77	4.87
	Electrocardiogram abnormal	7	2.89	1.58	3.74	7.39	3.52	15.53
	Hypertensive crisis	5	2.02	0.45	3.00	4.04	1.68	9.72
	Cardiotoxicity	4	2.06	0.30	3.14	4.18	1.57	11.14
	Electrocardiogram change	4	3.03	1.27	4.11	8.18	3.05	21.94
Tivozanib (4)	Blood pressure increased	35	3.58	3.01	3.98	11.94	8.52	16.72
	Dyspnoea	24	1.30	0.62	1.78	2.47	1.64	3.70
	Sinus tachycardia	4	2.68	0.92	3.76	6.42	2.41	17.15
	Blood pressure abnormal	3	2.08	0.01	3.28	4.22	1.36	13.11
Cediranib (3)	Hypertension	12	2.86	1.88	3.53	7.25	4.08	12.89
	Cerebrovascular accident	5	1.91	0.35	2.89	3.75	1.55	9.07
	Ejection fraction decreased	3	2.58	0.51	3.78	5.96	1.91	18.58
