# Supplementary material for: Cardiovascular toxicity associated with angiogenesis inhibitors: A comprehensive pharmacovigilance analysis based on the FDA Adverse Event Reporting System database from 2014 to 2021
Source: Front Cardiovasc Med. 2022 Oct 13;9:988013. doi: 10.3389/fcvm.2022.988013 (PMC9606330; doi:10.3389/fcvm.2022.988013)
Supplement: Supplementary file 4 [file Data_Sheet_4.PDF]

Drug	PT	N	IC	IC025	IC975	ROR	ROR025	ROR975
Bevacizumab (106)	Acute coronary syndrome	106	1.70	1.38	1.93	3.26	2.68	3.95
Aflibercept (47)	Acute coronary syndrome	19	3.10	2.33	3.64	8.57	5.46	13.45
Lenvatinib (35)	Acute coronary syndrome	18	1.16	0.37	1.71	2.24	1.41	3.55
Nintedanib (38)	Acute coronary syndrome	20	1.07	0.32	1.59	2.10	1.35	3.25
Sorafenib (37)	Acute coronary syndrome	20	1.35	0.60	1.88	2.55	1.65	3.96
Axitinib (24)	Acute coronary syndrome	12	1.25	0.27	1.91	2.37	1.35	4.18
Lenvatinib (35)	Acute myocardial infarction	51	0.91	0.44	1.24	1.87	1.42	2.47
Sorafenib (37)	Acute myocardial infarction	44	0.74	0.24	1.10	1.67	1.24	2.25
Aflibercept (47)	Angina pectoris	13	1.14	0.20	1.78	2.20	1.28	3.80
Regorafenib (23)	Angiotensin converting enzyme increased	3	2.57	0.50	3.77	5.92	1.86	18.83
Bevacizumab (106)	Aortic thrombosis	14	1.78	0.88	2.40	3.44	2.02	5.85
Aflibercept (47)	Aortic thrombosis	5	2.96	1.40	3.95	7.80	3.23	18.83
Lenvatinib (35)	Aortic thrombosis	5	1.98	0.42	2.96	3.94	1.63	9.50
Bevacizumab (106)	Arrhythmia supraventricular	33	3.12	2.54	3.53	8.69	6.10	12.38
Aflibercept (47)	Arterial occlusive disease	10	2.35	1.28	3.08	5.11	2.75	9.51
Bevacizumab (106)	Arterial thrombosis	34	2.18	1.61	2.59	4.53	3.22	6.38
Sorafenib (37)	Arterial thrombosis	6	1.59	0.17	2.50	3.01	1.35	6.71
Nintedanib (38)	Arteriosclerosis coronary artery	15	1.32	0.45	1.92	2.50	1.50	4.15
Bevacizumab (106)	Arteriospasm coronary	59	2.23	1.80	2.54	4.70	3.63	6.10
Apatinib (domestic) (35)	Arteriospasm coronary	8	3.01	1.80	3.81	8.06	4.02	16.14
Bevacizumab (106)	Ascites	738	2.76	2.63	2.84	6.76	6.27	7.28
Ramucirumab (25)	Ascites	113	4.33	4.02	4.55	20.08	16.67	24.18
Aflibercept (47)	Ascites	23	1.85	1.16	2.34	3.61	2.40	5.44
Sunitinib (27)	Ascites	245	2.15	1.94	2.30	4.43	3.91	5.03
Lenvatinib (35)	Ascites	198	2.89	2.66	3.06	7.42	6.45	8.54
Pazopanib (39)	Ascites	127	1.96	1.67	2.17	3.89	3.27	4.63
Cabozantinib (15)	Ascites	61	1.03	0.61	1.34	2.05	1.59	2.63
Sorafenib (37)	Ascites	373	3.85	3.68	3.97	14.41	13.00	15.97
Axitinib (24)	Ascites	33	1.01	0.43	1.42	2.02	1.43	2.84
Regorafenib (23)	Ascites	169	3.07	2.82	3.26	8.43	7.24	9.81
Apatinib (domestic) (35)	Ascites	30	2.48	1.87	2.91	5.59	3.90	8.00
Aflibercept (47)	Atrial fibrillation	36	0.85	0.29	1.24	1.80	1.30	2.49
Nintedanib (38)	Atrial fibrillation	141	0.45	0.17	0.65	1.36	1.15	1.61
Bevacizumab (106)	Atrial flutter	48	0.66	0.18	1.00	1.58	1.19	2.10
Aflibercept (47)	Atrial thrombosis	6	2.64	1.22	3.55	6.22	2.79	13.87
Nintedanib (38)	Atrioventricular block	18	1.17	0.37	1.72	2.24	1.41	3.56

Axitinib (24)	Autoimmune myocarditis	4	2.82	1.06	3.90	7.07	2.61	19.13
Bevacizumab (106)	Axillary vein thrombosis	11	2.60	1.58	3.29	6.06	3.30	11.14
Bevacizumab (106)	Blindness transient	55	1.02	0.57	1.34	2.03	1.56	2.65
Aflibercept (47)	Blindness transient	24	3.63	2.95	4.11	12.37	8.28	18.49
Aflibercept (47)	Blood creatine phosphokinase increased	22	2.10	1.39	2.60	4.30	2.83	6.53
Sorafenib (37)	Blood creatine phosphokinase MB increased	12	3.34	2.36	4.01	10.13	5.71	17.98
Sunitinib (27)	Blood pressure abnormal	69	0.83	0.44	1.12	1.78	1.41	2.26
Lenvatinib (35)	Blood pressure abnormal	73	1.96	1.57	2.24	3.89	3.09	4.90
Nintedanib (38)	Blood pressure abnormal	39	0.81	0.28	1.19	1.76	1.28	2.41
Pazopanib (39)	Blood pressure abnormal	48	1.07	0.59	1.41	2.10	1.58	2.79
Cabozantinib (15)	Blood pressure abnormal	54	1.36	0.91	1.69	2.57	1.97	3.36
Axitinib (24)	Blood pressure abnormal	41	1.82	1.30	2.19	3.52	2.59	4.78
Tivozanib (4)	Blood pressure abnormal	3	2.08	0.01	3.28	4.22	1.36	13.11
Bevacizumab (106)	Blood pressure diastolic abnormal	22	1.21	0.49	1.71	2.31	1.51	3.52
Sunitinib (27)	Blood pressure diastolic decreased	25	1.02	0.35	1.49	2.02	1.36	3.00
Bevacizumab (106)	Blood pressure diastolic increased	62	1.97	1.55	2.27	3.92	3.05	5.05
Lenvatinib (35)	Blood pressure diastolic increased	39	3.23	2.69	3.61	9.36	6.82	12.84
Axitinib (24)	Blood pressure diastolic increased	10	1.92	0.84	2.64	3.77	2.03	7.02
Regorafenib (23)	Blood pressure diastolic increased	9	1.53	0.39	2.30	2.89	1.50	5.57
Sunitinib (27)	Blood pressure fluctuation	83	0.65	0.29	0.91	1.57	1.27	1.95
Lenvatinib (35)	Blood pressure fluctuation	180	2.81	2.57	2.99	7.03	6.06	8.14
Cabozantinib (15)	Blood pressure fluctuation	88	1.62	1.26	1.87	3.07	2.49	3.78
Sorafenib (37)	Blood pressure fluctuation	38	0.63	0.09	1.01	1.55	1.12	2.13
Regorafenib (23)	Blood pressure fluctuation	31	0.70	0.11	1.13	1.63	1.15	2.32
Bevacizumab (106)	Blood pressure increased	1024	0.84	0.73	0.91	1.78	1.68	1.90
Sunitinib (27)	Blood pressure increased	1039	1.84	1.74	1.92	3.59	3.37	3.81
Lenvatinib (35)	Blood pressure increased	1418	3.35	3.27	3.42	10.22	9.69	10.78
Nintedanib (38)	Blood pressure increased	234	0.50	0.29	0.66	1.42	1.24	1.61
Pazopanib (39)	Blood pressure increased	656	1.94	1.82	2.04	3.85	3.56	4.16
Cabozantinib (15)	Blood pressure increased	935	2.58	2.47	2.66	5.99	5.61	6.39
Sorafenib (37)	Blood pressure increased	322	1.26	1.08	1.40	2.40	2.15	2.68
Axitinib (24)	Blood pressure increased	427	2.32	2.16	2.44	5.01	4.55	5.51
Regorafenib (23)	Blood pressure increased	346	1.74	1.56	1.87	3.34	3.00	3.71
Vandetanib (9)	Blood pressure increased	37	1.69	1.14	2.08	3.23	2.34	4.47
Tivozanib (4)	Blood pressure increased	35	3.58	3.01	3.98	11.94	8.52	16.72
Bevacizumab (106)	Blood pressure systolic increased	93	0.42	0.08	0.67	1.34	1.09	1.64
Sunitinib (27)	Blood pressure systolic increased	79	1.16	0.79	1.43	2.24	1.79	2.80

Lenvatinib (35)	Blood pressure systolic increased	32	0.92	0.33	1.33	1.89	1.33	2.67
Pazopanib (39)	Blood pressure systolic increased	36	0.79	0.24	1.19	1.73	1.25	2.40
Sorafenib (37)	Blood pressure systolic increased	30	0.87	0.26	1.30	1.83	1.28	2.61
Regorafenib (23)	Blood pressure systolic increased	29	1.18	0.56	1.62	2.27	1.58	3.27
Bevacizumab (106)	Brachiocephalic vein thrombosis	9	3.29	2.16	4.06	9.81	4.86	19.82
Pazopanib (39)	Brain natriuretic peptide increased	8	1.31	0.10	2.12	2.49	1.24	4.98
Aflibercept (47)	Cardiac aneurysm	3	2.34	0.27	3.55	5.07	1.63	15.77
Apatinib (domestic) (35)	Cardiac arrest	22	0.73	0.01	1.23	1.66	1.09	2.52
Bevacizumab (106)	Cardiac dysfunction	28	1.22	0.58	1.66	2.32	1.60	3.37
Lenvatinib (35)	Cardiac dysfunction	16	2.30	1.46	2.89	4.94	3.02	8.09
Pazopanib (39)	Cardiac dysfunction	16	2.05	1.21	2.63	4.13	2.53	6.76
Axitinib (24)	Cardiac dysfunction	13	2.59	1.65	3.23	6.02	3.49	10.39
Bevacizumab (106)	Cardiac failure	445	0.59	0.43	0.70	1.50	1.37	1.65
Ramucirumab (25)	Cardiac failure	51	1.82	1.36	2.16	3.54	2.69	4.66
Aflibercept (47)	Cardiac failure	45	1.43	0.94	1.79	2.70	2.02	3.62
Lenvatinib (35)	Cardiac failure	128	0.84	0.54	1.05	1.79	1.50	2.12
Nintedanib (38)	Cardiac failure	113	0.40	0.09	0.63	1.32	1.10	1.59
Pazopanib (39)	Cardiac failure	154	0.81	0.54	1.00	1.75	1.49	2.05
Axitinib (24)	Cardiac failure	84	0.93	0.57	1.19	1.90	1.54	2.36
Apatinib (domestic) (35)	Cardiac failure	23	0.75	0.05	1.24	1.68	1.12	2.53
Bevacizumab (106)	Cardiac failure acute	47	0.96	0.47	1.30	1.94	1.46	2.59
Ramucirumab (25)	Cardiac failure acute	6	1.99	0.58	2.90	3.98	1.78	8.86
Pazopanib (39)	Cardiac failure acute	19	1.36	0.59	1.90	2.56	1.63	4.03
Sorafenib (37)	Cardiac failure acute	14	1.25	0.35	1.87	2.38	1.41	4.02
Axitinib (24)	Cardiac failure acute	10	1.38	0.30	2.10	2.60	1.40	4.83
Aflibercept (47)	Cardiac failure chronic	5	1.95	0.39	2.94	3.87	1.61	9.30
Apatinib (domestic) (35)	Cardiac tamponade	5	2.07	0.51	3.05	4.19	1.74	10.09
Pazopanib (39)	Cardiac ventricular scarring	3	2.74	0.67	3.95	6.69	1.77	25.22
Bevacizumab (106)	Cardiac ventricular thrombosis	19	2.19	1.42	2.72	4.55	2.88	7.19
Aflibercept (47)	Cardiac ventricular thrombosis	13	4.25	3.31	4.89	19.05	10.98	33.03
Pazopanib (39)	Cardiac ventricular thrombosis	5	1.76	0.20	2.75	3.39	1.40	8.18
Sorafenib (37)	Cardiac ventricular thrombosis	5	1.99	0.43	2.97	3.97	1.65	9.58
Apatinib (domestic) (35)	Cardiomegaly	6	1.53	0.11	2.44	2.88	1.29	6.42
Bevacizumab (106)	Cardiomyopathy	110	1.10	0.78	1.33	2.14	1.78	2.59
Pazopanib (39)	Cardiomyopathy	37	1.26	0.71	1.65	2.40	1.74	3.31
Sorafenib (37)	Cardiomyopathy	21	0.79	0.06	1.30	1.73	1.13	2.65
Apatinib (domestic) (35)	Cardiomyopathy	12	2.14	1.17	2.81	4.42	2.51	7.79

Sunitinib (27)	Cardiopulmonary failure	94	3.61	3.27	3.86	12.22	9.94	15.03
Bevacizumab (106)	Cardiotoxicity	109	1.85	1.54	2.08	3.61	2.99	4.37
Sunitinib (27)	Cardiotoxicity	25	0.72	0.05	1.19	1.64	1.11	2.43
Pazopanib (39)	Cardiotoxicity	27	1.56	0.92	2.01	2.95	2.02	4.30
Sorafenib (37)	Cardiotoxicity	30	2.03	1.42	2.46	4.09	2.85	5.85
Apatinib (domestic) (35)	Cardiotoxicity	35	4.25	3.69	4.65	19.02	13.63	26.54
Vandetanib (9)	Cardiotoxicity	4	2.06	0.30	3.14	4.18	1.57	11.14
Bevacizumab (106)	Catheter site thrombosis	7	2.46	1.16	3.31	5.49	2.56	11.78
Bevacizumab (106)	Cerebral infarction	202	1.10	0.87	1.27	2.15	1.87	2.47
Ramucirumab (25)	Cerebral infarction	15	1.65	0.78	2.25	3.14	1.89	5.21
Aflibercept (47)	Cerebral infarction	25	2.17	1.50	2.64	4.50	3.04	6.66
Lenvatinib (35)	Cerebral infarction	66	1.53	1.12	1.82	2.88	2.26	3.67
Nintedanib (38)	Cerebral infarction	52	0.93	0.47	1.26	1.91	1.45	2.51
Sorafenib (37)	Cerebral infarction	56	1.33	0.89	1.65	2.52	1.94	3.28
Axitinib (24)	Cerebral infarction	42	1.56	1.05	1.93	2.96	2.18	4.01
Regorafenib (23)	Cerebral infarction	34	0.99	0.42	1.39	1.98	1.42	2.78
Bevacizumab (106)	Cerebral ischaemia	99	2.49	2.15	2.73	5.60	4.58	6.85
Lenvatinib (35)	Cerebrovascular accident	192	0.39	0.15	0.56	1.31	1.14	1.51
Nintedanib (38)	Cerebrovascular accident	216	0.31	0.08	0.47	1.24	1.08	1.41
Axitinib (24)	Cerebrovascular accident	112	0.32	0.01	0.54	1.25	1.04	1.50
Cediranib (3)	Cerebrovascular accident	5	1.91	0.35	2.89	3.75	1.55	9.07
Bevacizumab (106)	Cerebrovascular disorder	22	1.33	0.61	1.83	2.51	1.64	3.82
Lenvatinib (35)	Chest pain	194	0.29	0.06	0.46	1.22	1.06	1.41
Nintedanib (38)	Chest pain	346	0.87	0.69	1.00	1.83	1.64	2.03
Bevacizumab (106)	Choroidal infarction	6	2.63	1.21	3.54	6.17	2.68	14.20
Bevacizumab (106)	Collateral circulation	6	2.57	1.15	3.48	5.93	2.59	13.61
Bevacizumab (106)	Computerised tomogram thorax abnormal	14	1.76	0.86	2.38	3.39	2.00	5.77
Pazopanib (39)	Computerised tomogram thorax abnormal	8	2.39	1.18	3.20	5.25	2.61	10.56
Apatinib (domestic) (35)	Computerised tomogram thorax abnormal	7	3.48	2.17	4.33	11.13	5.28	23.47
Bevacizumab (106)	Congestive cardiomyopathy	51	1.51	1.04	1.84	2.85	2.16	3.75
Aflibercept (47)	Congestive cardiomyopathy	5	1.90	0.34	2.89	3.74	1.55	8.99
Regorafenib (23)	Congestive cardiomyopathy	15	2.08	1.21	2.68	4.22	2.54	7.01
Nintedanib (38)	Cor pulmonale	5	1.86	0.29	2.84	3.62	1.50	8.74
Bevacizumab (106)	Coronary artery stenosis	34	1.31	0.74	1.72	2.48	1.77	3.48
Bevacizumab (106)	Deep vein thrombosis	679	1.46	1.33	1.55	2.74	2.54	2.96
Ramucirumab (25)	Deep vein thrombosis	36	1.58	1.03	1.98	2.99	2.16	4.15
Aflibercept (47)	Deep vein thrombosis	41	1.55	1.03	1.93	2.94	2.16	3.99

Apatinib (domestic) (35)	Deep vein thrombosis	27	1.23	0.58	1.68	2.34	1.60	3.41
Vandetanib (9)	Deep vein thrombosis	15	1.55	0.68	2.15	2.93	1.77	4.87
Bevacizumab (106)	Device related thrombosis	26	2.61	1.95	3.07	6.10	4.11	9.05
Apatinib (domestic) (35)	Diastolic dysfunction	5	2.50	0.94	3.49	5.66	2.35	13.63
Bevacizumab (106)	Disseminated intravascular coagulation	209	2.20	1.97	2.37	4.60	4.01	5.28
Ramucirumab (25)	Disseminated intravascular coagulation	51	4.29	3.83	4.62	19.57	14.84	25.79
Sunitinib (27)	Disseminated intravascular coagulation	77	1.74	1.36	2.01	3.34	2.67	4.18
Lenvatinib (35)	Disseminated intravascular coagulation	22	0.99	0.27	1.49	1.98	1.30	3.01
Pazopanib (39)	Disseminated intravascular coagulation	71	2.37	1.97	2.65	5.16	4.09	6.52
Sorafenib (37)	Disseminated intravascular coagulation	22	1.03	0.32	1.53	2.04	1.34	3.10
Regorafenib (23)	Disseminated intravascular coagulation	62	2.86	2.44	3.16	7.26	5.65	9.32
Nintedanib (38)	Dizziness exertional	6	2.39	0.97	3.30	5.23	2.33	11.71
Nintedanib (38)	Dyspnoea	1854	1.56	1.48	1.61	2.94	2.81	3.08
Pazopanib (39)	Dyspnoea	720	0.15	0.03	0.24	1.11	1.03	1.19
Tivozanib (4)	Dyspnoea	24	1.30	0.62	1.78	2.47	1.64	3.70
Nintedanib (38)	Dyspnoea at rest	14	1.83	0.93	2.45	3.56	2.10	6.02
Regorafenib (23)	Dyspnoea at rest	7	1.45	0.15	2.31	2.74	1.30	5.76
Apatinib (domestic) (35)	Dyspnoea at rest	6	2.61	1.19	3.52	6.10	2.73	13.59
Bevacizumab (106)	Dyspnoea exertional	238	0.74	0.53	0.90	1.67	1.47	1.90
Nintedanib (38)	Dyspnoea exertional	408	3.30	3.14	3.42	9.85	8.93	10.87
Regorafenib (23)	Dyspnoea exertional	58	1.16	0.73	1.48	2.24	1.73	2.90
Apatinib (domestic) (35)	Ejection fraction abnormal	11	3.99	2.97	4.69	15.91	8.77	28.87
Bevacizumab (106)	Ejection fraction decreased	165	1.59	1.33	1.78	3.01	2.58	3.51
Aflibercept (47)	Ejection fraction decreased	20	2.55	1.80	3.07	5.85	3.77	9.07
Sunitinib (27)	Ejection fraction decreased	65	1.23	0.81	1.52	2.34	1.83	2.99
Lenvatinib (35)	Ejection fraction decreased	28	1.06	0.43	1.51	2.09	1.44	3.03
Pazopanib (39)	Ejection fraction decreased	76	2.20	1.82	2.47	4.59	3.66	5.75
Cabozantinib (15)	Ejection fraction decreased	29	0.95	0.33	1.39	1.93	1.34	2.78
Sorafenib (37)	Ejection fraction decreased	25	0.95	0.28	1.42	1.93	1.30	2.85
Axitinib (24)	Ejection fraction decreased	17	1.03	0.21	1.60	2.04	1.27	3.29
Regorafenib (23)	Ejection fraction decreased	20	0.99	0.24	1.51	1.98	1.28	3.08
Apatinib (domestic) (35)	Ejection fraction decreased	70	4.55	4.16	4.84	23.48	18.54	29.73
Cediranib (3)	Ejection fraction decreased	3	2.58	0.51	3.78	5.96	1.91	18.58
Vandetanib (9)	Electrocardiogram abnormal	7	2.89	1.58	3.74	7.39	3.52	15.53
Pazopanib (39)	Electrocardiogram change	4	1.83	0.07	2.91	3.56	1.33	9.54
Sorafenib (37)	Electrocardiogram change	6	2.56	1.15	3.47	5.90	2.63	13.22
Vandetanib (9)	Electrocardiogram change	4	3.03	1.27	4.11	8.18	3.05	21.94

Apatinib (domestic) (35)	Electrocardiogram QT prolonged	28	2.06	1.43	2.51	4.18	2.88	6.06
Vandetanib (9)	Electrocardiogram QT prolonged	87	4.78	4.42	5.04	27.47	22.21	33.98
Bevacizumab (106)	Electrocardiogram ST segment depression	16	1.14	0.30	1.73	2.21	1.35	3.62
Pazopanib (39)	Electrocardiogram T wave inversion	8	1.70	0.49	2.50	3.24	1.62	6.51
Bevacizumab (106)	Embolism	333	3.41	3.23	3.54	10.63	9.50	11.88
Ramucirumab (25)	Embolism	7	1.93	0.63	2.79	3.82	1.82	8.02
Aflibercept (47)	Embolism	10	2.25	1.18	2.98	4.77	2.56	8.86
Lenvatinib (35)	Embolism	19	1.29	0.52	1.83	2.45	1.56	3.85
Nintedanib (38)	Embolism	21	1.19	0.46	1.71	2.29	1.49	3.51
Cabozantinib (15)	Embolism	41	2.22	1.70	2.59	4.67	3.43	6.35
Regorafenib (23)	Embolism	13	1.15	0.22	1.80	2.22	1.29	3.83
Apatinib (domestic) (35)	Embolism	6	1.77	0.35	2.68	3.41	1.53	7.59
Bevacizumab (106)	Embolism arterial	37	2.84	2.29	3.23	7.15	5.13	9.96
Aflibercept (47)	Embolism arterial	7	3.30	2.00	4.15	9.85	4.68	20.74
Nintedanib (38)	Embolism arterial	6	1.80	0.39	2.71	3.48	1.56	7.78
Bevacizumab (106)	Embolism venous	122	3.16	2.86	3.38	8.94	7.44	10.74
Ramucirumab (25)	Embolism venous	10	3.22	2.14	3.95	9.33	5.01	17.38
Nintedanib (38)	Embolism venous	13	1.65	0.71	2.29	3.14	1.82	5.41
Axitinib (24)	Essential hypertension	7	1.93	0.62	2.78	3.80	1.81	7.99
Aflibercept (47)	Haemorrhagic stroke	11	2.24	1.21	2.93	4.71	2.61	8.52
Lenvatinib (35)	Haemorrhagic stroke	16	0.88	0.03	1.46	1.84	1.12	3.00
Nintedanib (38)	Heart rate abnormal	14	1.13	0.23	1.75	2.19	1.30	3.70
Bevacizumab (106)	Hemiparesis	219	1.73	1.51	1.90	3.33	2.91	3.81
Vandetanib (9)	Hemiparesis	20	3.54	2.79	4.06	11.60	7.47	18.01
Bevacizumab (106)	Hemiplegia	77	1.29	0.91	1.56	2.44	1.95	3.06
Sorafenib (37)	Hepatic artery thrombosis	3	2.27	0.20	3.47	4.81	1.53	15.12
Bevacizumab (106)	Hepatic infarction	5	1.60	0.04	2.59	3.04	1.25	7.38
Sorafenib (37)	Hepatic infarction	3	2.12	0.05	3.32	4.34	1.39	13.58
Sorafenib (37)	Hepatomegaly	29	1.84	1.22	2.28	3.58	2.48	5.15
Regorafenib (23)	Hepatomegaly	17	1.43	0.62	2.00	2.70	1.68	4.35
Bevacizumab (106)	Hypertension	3546	2.21	2.15	2.25	4.62	4.46	4.77
Ramucirumab (25)	Hypertension	144	1.97	1.69	2.17	3.92	3.32	4.62
Aflibercept (47)	Hypertension	309	2.85	2.66	2.99	7.22	6.45	8.09
Sunitinib (27)	Hypertension	1340	1.79	1.70	1.85	3.46	3.27	3.65
Lenvatinib (35)	Hypertension	1585	3.09	3.01	3.15	8.54	8.12	8.98
Nintedanib (38)	Hypertension	312	0.50	0.31	0.63	1.41	1.26	1.58
Pazopanib (39)	Hypertension	1103	2.27	2.17	2.35	4.83	4.55	5.13

Cabozantinib (15)	Hypertension	701	1.75	1.62	1.84	3.36	3.11	3.62
Sorafenib (37)	Hypertension	549	1.61	1.47	1.71	3.05	2.81	3.32
Axitinib (24)	Hypertension	750	2.72	2.60	2.80	6.58	6.11	7.07
Regorafenib (23)	Hypertension	553	1.99	1.85	2.10	3.98	3.66	4.33
Vandetanib (9)	Hypertension	57	1.90	1.46	2.22	3.74	2.88	4.86
Cediranib (3)	Hypertension	12	2.86	1.88	3.53	7.25	4.08	12.89
Bevacizumab (106)	Hypertensive crisis	182	2.02	1.78	2.20	4.06	3.51	4.71
Aflibercept (47)	Hypertensive crisis	36	3.63	3.07	4.02	12.37	8.91	17.18
Sunitinib (27)	Hypertensive crisis	51	1.17	0.70	1.50	2.25	1.71	2.96
Lenvatinib (35)	Hypertensive crisis	41	1.89	1.37	2.26	3.70	2.72	5.03
Pazopanib (39)	Hypertensive crisis	66	2.28	1.87	2.58	4.86	3.82	6.20
Axitinib (24)	Hypertensive crisis	18	1.39	0.59	1.94	2.62	1.65	4.15
Regorafenib (23)	Hypertensive crisis	17	1.04	0.22	1.61	2.06	1.28	3.31
Vandetanib (9)	Hypertensive crisis	5	2.02	0.45	3.00	4.04	1.68	9.72
Lenvatinib (35)	Hypertensive emergency	6	2.16	0.75	3.07	4.47	2.00	9.99
Regorafenib (23)	Hypertensive emergency	4	1.89	0.13	2.97	3.71	1.39	9.92
Bevacizumab (106)	Hypertensive encephalopathy	18	2.98	2.18	3.53	7.87	4.88	12.70
Lenvatinib (35)	Hypertensive encephalopathy	4	2.25	0.48	3.33	4.75	1.77	12.77
Pazopanib (39)	Hypertensive encephalopathy	6	2.63	1.22	3.54	6.20	2.76	13.92
Axitinib (24)	Hypertensive encephalopathy	4	2.54	0.77	3.62	5.81	2.17	15.61
Bevacizumab (106)	Hypertensive urgency	6	1.89	0.48	2.81	3.72	1.65	8.39
Lenvatinib (35)	Hypertensive urgency	5	2.78	1.22	3.76	6.87	2.82	16.71
Cabozantinib (15)	Hypertensive urgency	4	2.42	0.66	3.50	5.36	1.99	14.46
Bevacizumab (106)	Hypoxia	217	0.81	0.59	0.97	1.76	1.54	2.01
Ramucirumab (25)	Hypoxia	14	1.19	0.29	1.81	2.29	1.35	3.86
Nintedanib (38)	Hypoxia	120	1.74	1.44	1.96	3.34	2.79	4.00
Apatinib (domestic) (35)	Hypoxia	16	1.43	0.59	2.01	2.70	1.65	4.40
Axitinib (24)	Immune-mediated myocarditis	3	2.29	0.22	3.50	4.90	1.57	15.33
Bevacizumab (106)	Intestinal infarction	8	1.29	0.08	2.10	2.45	1.22	4.93
Bevacizumab (106)	Ischaemic stroke	116	0.59	0.28	0.81	1.50	1.25	1.80
Aflibercept (47)	Ischaemic stroke	12	1.40	0.42	2.06	2.63	1.49	4.63
Bevacizumab (106)	Jugular vein thrombosis	29	2.15	1.53	2.58	4.42	3.05	6.41
Ramucirumab (25)	Jugular vein thrombosis	6	3.04	1.62	3.95	8.21	3.68	18.32
Aflibercept (47)	Jugular vein thrombosis	7	3.16	1.86	4.01	8.93	4.24	18.78
Bevacizumab (106)	Labile blood pressure	11	1.34	0.32	2.04	2.53	1.39	4.60
Bevacizumab (106)	Lacunar infarction	19	1.31	0.54	1.85	2.48	1.57	3.90
Apatinib (domestic) (35)	Lacunar infarction	4	2.43	0.66	3.51	5.38	2.01	14.36

Aflibercept (47)	Left atrial dilatation	3	2.24	0.17	3.45	4.73	1.52	14.71
Pazopanib (39)	Left atrial dilatation	5	1.62	0.06	2.60	3.07	1.27	7.40
Aflibercept (47)	Left ventricular dilatation	4	2.83	1.07	3.91	7.12	2.66	19.08
Bevacizumab (106)	Left ventricular dysfunction	96	1.91	1.57	2.15	3.75	3.06	4.59
Ramucirumab (25)	Left ventricular dysfunction	5	1.70	0.14	2.68	3.24	1.35	7.80
Aflibercept (47)	Left ventricular dysfunction	8	2.17	0.95	2.97	4.49	2.24	8.99
Pazopanib (39)	Left ventricular dysfunction	34	2.11	1.54	2.52	4.32	3.08	6.05
Sorafenib (37)	Left ventricular dysfunction	13	1.08	0.14	1.72	2.11	1.22	3.64
Apatinib (domestic) (35)	Left ventricular dysfunction	17	3.40	2.59	3.97	10.58	6.57	17.04
Apatinib (domestic) (35)	Left ventricular enlargement	3	2.66	0.59	3.86	6.30	2.01	19.74
Bevacizumab (106)	Left ventricular failure	23	1.01	0.31	1.50	2.02	1.34	3.05
Apatinib (domestic) (35)	Left ventricular failure	9	3.23	2.09	4.00	9.41	4.88	18.12
Bevacizumab (106)	Lung opacity	20	1.66	0.91	2.18	3.15	2.02	4.91
Bevacizumab (106)	Malignant hypertension	17	2.24	1.42	2.80	4.71	2.90	7.65
Aflibercept (47)	Malignant hypertension	3	2.37	0.30	3.58	5.17	1.66	16.09
Sunitinib (27)	Malignant hypertension	6	1.62	0.20	2.53	3.07	1.37	6.86
Pazopanib (39)	Malignant hypertension	5	1.92	0.36	2.91	3.79	1.57	9.14
Bevacizumab (106)	Mental status changes	179	0.93	0.68	1.11	1.91	1.64	2.21
Lenvatinib (35)	Mental status changes	35	0.62	0.06	1.02	1.54	1.10	2.14
Sunitinib (27)	Mesenteric artery stenosis	3	2.35	0.28	3.56	5.10	1.59	16.37
Cabozantinib (15)	Mesenteric artery stenosis	3	2.54	0.47	3.75	5.83	1.82	18.71
Aflibercept (47)	Mesenteric artery thrombosis	4	2.96	1.19	4.03	7.76	2.88	20.87
Bevacizumab (106)	Mesenteric vein thrombosis	16	2.02	1.18	2.61	4.06	2.47	6.69
Nintedanib (38)	Mesenteric vein thrombosis	14	3.25	2.35	3.87	9.51	5.59	16.17
Sorafenib (37)	Mesenteric vein thrombosis	7	2.49	1.19	3.35	5.63	2.67	11.88
Regorafenib (23)	Mesenteric vein thrombosis	4	1.98	0.21	3.06	3.94	1.47	10.54
Bevacizumab (106)	Monoparesis	17	1.30	0.48	1.87	2.46	1.52	3.97
Sunitinib (27)	Monoplegia	16	0.96	0.12	1.54	1.95	1.19	3.18
Pazopanib (39)	Monoplegia	11	1.15	0.12	1.84	2.22	1.23	4.01
Nintedanib (38)	Myocardial injury	3	2.62	0.55	3.83	6.15	1.88	20.11
Bevacizumab (106)	Myocardial ischaemia	79	1.03	0.66	1.30	2.04	1.63	2.55
Sorafenib (37)	Myocardial ischaemia	18	0.96	0.17	1.52	1.95	1.23	3.10
Apatinib (domestic) (35)	Myocardial ischaemia	6	1.53	0.11	2.44	2.89	1.30	6.43
Aflibercept (47)	Myocardial necrosis	4	3.00	1.23	4.08	7.99	2.96	21.57
Bevacizumab (106)	Myocarditis	61	0.77	0.34	1.07	1.70	1.32	2.19
Axitinib (24)	Myocarditis	33	2.54	1.96	2.95	5.81	4.12	8.18
Ramucirumab (25)	Nocturnal dyspnoea	5	2.90	1.34	3.88	7.45	3.09	17.97

Bevacizumab (106)	Oedema	298	0.61	0.42	0.75	1.52	1.36	1.71
Ramucirumab (25)	Oedema	46	2.25	1.76	2.60	4.77	3.57	6.37
Sunitinib (27)	Oedema	160	0.70	0.43	0.89	1.62	1.39	1.89
Lenvatinib (35)	Oedema	77	0.70	0.32	0.97	1.63	1.30	2.04
Apatinib (domestic) (35)	Oedema	21	1.19	0.46	1.71	2.29	1.49	3.51
Bevacizumab (106)	Oedema peripheral	565	0.65	0.52	0.76	1.57	1.45	1.71
Ramucirumab (25)	Oedema peripheral	38	1.14	0.60	1.52	2.20	1.60	3.03
Aflibercept (47)	Oedema peripheral	34	0.77	0.19	1.17	1.70	1.21	2.38
Sunitinib (27)	Oedema peripheral	292	0.69	0.50	0.83	1.61	1.44	1.81
Lenvatinib (35)	Oedema peripheral	161	0.89	0.63	1.08	1.86	1.59	2.17
Pazopanib (39)	Oedema peripheral	155	0.54	0.27	0.73	1.45	1.24	1.70
Sorafenib (37)	Oedema peripheral	165	0.97	0.71	1.16	1.96	1.68	2.29
Regorafenib (23)	Oedema peripheral	115	0.83	0.52	1.05	1.77	1.48	2.13
Apatinib (domestic) (35)	Oedema peripheral	27	0.71	0.07	1.16	1.63	1.12	2.38
Pazopanib (39)	Orthopnoea	15	1.95	1.08	2.56	3.87	2.33	6.44
Apatinib (domestic) (35)	Orthopnoea	5	2.40	0.84	3.39	5.30	2.20	12.74
Aflibercept (47)	Orthostatic hypotension	11	1.55	0.53	2.25	2.94	1.62	5.30
Nintedanib (38)	Paraparesis	9	1.95	0.81	2.71	3.85	2.00	7.42
Pazopanib (39)	Paresis	9	1.73	0.60	2.50	3.33	1.73	6.41
Nintedanib (38)	Paroxysmal arrhythmia	3	2.54	0.47	3.75	5.83	1.81	18.75
Bevacizumab (106)	Pelvic venous thrombosis	21	1.75	1.02	2.27	3.37	2.19	5.20
Axitinib (24)	Pericardial disease	3	2.43	0.36	3.64	5.40	1.72	16.96
Bevacizumab (106)	Pericardial effusion	149	0.86	0.59	1.06	1.82	1.55	2.14
Sunitinib (27)	Pericardial effusion	111	1.42	1.11	1.65	2.68	2.22	3.23
Pazopanib (39)	Pericardial effusion	52	1.09	0.63	1.42	2.13	1.62	2.79
Apatinib (domestic) (35)	Pericardial effusion	33	2.99	2.41	3.40	7.93	5.63	11.17
Apatinib (domestic) (35)	Pericarditis	9	1.96	0.82	2.72	3.89	2.02	7.49
Bevacizumab (106)	Peripheral arterial occlusive disease	27	0.67	0.02	1.12	1.59	1.09	2.32
Pazopanib (39)	Peripheral artery occlusion	7	1.63	0.33	2.49	3.10	1.47	6.53
Nintedanib (38)	Peripheral artery thrombosis	8	1.79	0.58	2.60	3.46	1.73	6.95
Sunitinib (27)	Peripheral swelling	464	0.39	0.24	0.50	1.31	1.20	1.44
Lenvatinib (35)	Peripheral swelling	224	0.40	0.18	0.56	1.32	1.16	1.51
Cabozantinib (15)	Peripheral swelling	225	0.24	0.02	0.40	1.18	1.04	1.35
Sorafenib (37)	Peripheral swelling	239	0.54	0.33	0.70	1.46	1.28	1.65
Lenvatinib (35)	Portal vein occlusion	4	2.86	1.10	3.94	7.26	2.65	19.92
Sorafenib (37)	Portal vein occlusion	3	2.51	0.44	3.71	5.68	1.79	18.07
Bevacizumab (106)	Portal vein thrombosis	74	2.62	2.23	2.89	6.13	4.85	7.74

Ramucirumab (25)	Portal vein thrombosis	9	3.17	2.04	3.94	9.03	4.69	17.39
Aflibercept (47)	Portal vein thrombosis	4	1.98	0.21	3.06	3.94	1.48	10.50
Lenvatinib (35)	Portal vein thrombosis	39	3.58	3.04	3.96	11.93	8.68	16.39
Nintedanib (38)	Portal vein thrombosis	13	1.81	0.87	2.45	3.50	2.03	6.04
Cabozantinib (15)	Portal vein thrombosis	9	1.37	0.23	2.14	2.59	1.35	4.99
Sorafenib (37)	Portal vein thrombosis	46	3.85	3.36	4.20	14.42	10.76	19.34
Lenvatinib (35)	Portopulmonary hypertension	3	2.52	0.45	3.72	5.73	1.80	18.24
Nintedanib (38)	Pulmonary arterial hypertension	39	0.90	0.36	1.28	1.86	1.36	2.55
Bevacizumab (106)	Pulmonary artery thrombosis	25	2.99	2.32	3.46	7.93	5.29	11.89
Ramucirumab (25)	Pulmonary artery thrombosis	3	2.48	0.41	3.68	5.57	1.79	17.34
Bevacizumab (106)	Pulmonary embolism	1037	1.59	1.49	1.66	3.01	2.83	3.20
Ramucirumab (25)	Pulmonary embolism	63	1.92	1.50	2.22	3.77	2.95	4.84
Aflibercept (47)	Pulmonary embolism	105	2.44	2.11	2.67	5.41	4.46	6.55
Lenvatinib (35)	Pulmonary embolism	198	1.25	1.01	1.42	2.37	2.06	2.73
Nintedanib (38)	Pulmonary embolism	190	0.93	0.69	1.11	1.91	1.66	2.20
Cabozantinib (15)	Pulmonary embolism	236	1.33	1.11	1.49	2.51	2.21	2.86
Apatinib (domestic) (35)	Pulmonary embolism	33	1.05	0.47	1.46	2.07	1.47	2.91
Nintedanib (38)	Pulmonary hypertension	116	2.30	1.99	2.52	4.91	4.09	5.90
Bevacizumab (106)	Pulmonary infarction	15	0.88	0.01	1.48	1.84	1.11	3.07
Axitinib (24)	Pulmonary infarction	5	1.72	0.16	2.70	3.29	1.37	7.92
Sunitinib (27)	Pulmonary oedema	170	1.03	0.78	1.21	2.04	1.76	2.38
Axitinib (24)	Pulmonary oedema	46	0.90	0.41	1.25	1.87	1.40	2.50
Bevacizumab (106)	Pulmonary thrombosis	60	0.48	0.05	0.79	1.40	1.08	1.80
Sunitinib (27)	Pulmonary thrombosis	41	0.91	0.39	1.28	1.88	1.38	2.55
Nintedanib (38)	Pulmonary thrombosis	31	1.29	0.70	1.72	2.45	1.72	3.49
Bevacizumab (106)	Pulmonary tumour thrombotic microangiopathy	5	2.86	1.30	3.85	7.28	2.83	18.73
Bevacizumab (106)	Pulmonary valve incompetence	11	1.43	0.41	2.13	2.70	1.48	4.90
Cabozantinib (15)	Pulmonary venous thrombosis	3	2.39	0.33	3.60	5.26	1.66	16.64
Nintedanib (38)	Pulseless electrical activity	26	2.22	1.56	2.68	4.65	3.16	6.84
Bevacizumab (106)	Renal artery occlusion	4	1.77	0.00	2.85	3.40	1.26	9.22
Pazopanib (39)	Renal artery occlusion	3	2.23	0.16	3.44	4.70	1.49	14.81
Pazopanib (39)	Renal infarct	6	1.61	0.19	2.52	3.05	1.36	6.80
Sunitinib (27)	Renal vein thrombosis	5	1.80	0.24	2.79	3.49	1.44	8.44
Bevacizumab (106)	Retinal artery occlusion	20	1.20	0.44	1.72	2.29	1.47	3.56
Aflibercept (47)	Retinal artery occlusion	11	3.57	2.55	4.27	11.90	6.57	21.56
Bevacizumab (106)	Retinal vein occlusion	42	2.15	1.64	2.52	4.43	3.26	6.03
Aflibercept (47)	Retinal vein occlusion	11	3.52	2.50	4.22	11.49	6.34	20.80

Sorafenib (37)	Retinal vein occlusion	8	1.70	0.48	2.50	3.24	1.62	6.50
Sorafenib (37)	Retinal vein thrombosis	6	2.76	1.35	3.67	6.79	3.02	15.25
Bevacizumab (106)	Retinopathy hypertensive	7	2.35	1.05	3.20	5.10	2.38	10.90
Lenvatinib (35)	Rhythm idioventricular	10	3.79	2.71	4.51	13.79	7.26	26.17
Bevacizumab (106)	Right ventricular dilatation	9	1.59	0.45	2.35	3.01	1.55	5.83
Nintedanib (38)	Right ventricular failure	19	1.03	0.26	1.57	2.05	1.31	3.22
Bevacizumab (106)	Secondary hypertension	9	2.54	1.40	3.30	5.81	2.97	11.39
Sorafenib (37)	Secondary hypertension	4	2.56	0.79	3.64	5.88	2.18	15.87
Apatinib (domestic) (35)	Secondary hypertension	7	3.76	2.46	4.61	13.54	6.35	28.87
Lenvatinib (35)	Sinus arrest	11	2.72	1.69	3.41	6.58	3.63	11.94
Bevacizumab (106)	Sinus tachycardia	78	0.74	0.36	1.01	1.67	1.34	2.09
Tivozanib (4)	Sinus tachycardia	4	2.68	0.92	3.76	6.42	2.41	17.15
Bevacizumab (106)	Splenic artery thrombosis	7	3.30	2.00	4.15	9.84	4.32	22.40
Bevacizumab (106)	Splenic infarction	16	1.23	0.39	1.81	2.34	1.43	3.84
Aflibercept (47)	Splenic thrombosis	3	2.71	0.64	3.91	6.53	2.07	20.62
Nintedanib (38)	Splenic vein thrombosis	10	3.33	2.26	4.06	10.08	5.36	18.97
Bevacizumab (106)	Stress cardiomyopathy	46	1.10	0.61	1.45	2.15	1.61	2.88
Bevacizumab (106)	Subclavian vein thrombosis	18	1.92	1.13	2.47	3.78	2.37	6.05
Aflibercept (47)	Subclavian vein thrombosis	6	3.13	1.72	4.04	8.76	3.92	19.58
Apatinib (domestic) (35)	Subclavian vein thrombosis	4	2.68	0.92	3.76	6.42	2.40	17.16
Bevacizumab (106)	Sudden death	82	1.24	0.88	1.51	2.37	1.90	2.95
Ramucirumab (25)	Sudden death	11	2.44	1.41	3.13	5.41	2.99	9.78
Aflibercept (47)	Sudden death	11	2.26	1.24	2.96	4.81	2.66	8.69
Sunitinib (27)	Sudden death	28	0.68	0.05	1.12	1.60	1.10	2.32
Lenvatinib (35)	Sudden death	18	1.08	0.28	1.63	2.11	1.33	3.35
Cabozantinib (15)	Sudden death	21	1.13	0.40	1.64	2.19	1.43	3.36
Axitinib (24)	Sudden death	16	1.56	0.72	2.15	2.96	1.81	4.83
Bevacizumab (106)	Superior vena cava syndrome	36	3.44	2.88	3.83	10.83	7.69	15.24
Aflibercept (47)	Superior vena cava syndrome	8	3.69	2.48	4.49	12.91	6.41	26.00
Apatinib (domestic) (35)	Superior vena cava syndrome	13	4.41	3.47	5.05	21.27	12.24	36.95
Sorafenib (37)	Systolic dysfunction	5	1.76	0.20	2.74	3.38	1.40	8.15
Sorafenib (37)	Systolic hypertension	8	3.29	2.07	4.09	9.76	4.82	19.76
Regorafenib (23)	Systolic hypertension	5	2.81	1.24	3.79	6.99	2.88	16.97
Bevacizumab (106)	Tachycardia	366	0.22	0.05	0.35	1.17	1.05	1.29
Aflibercept (47)	Tachycardia	33	0.91	0.33	1.33	1.88	1.34	2.65
Nintedanib (38)	Thrombolysis	3	2.08	0.01	3.28	4.22	1.35	13.23
Bevacizumab (106)	Thrombophlebitis	41	1.64	1.12	2.01	3.11	2.28	4.24

Bevacizumab (106)	Thrombophlebitis migrans	18	3.91	3.12	4.46	15.04	9.04	25.00
Bevacizumab (106)	Thrombosis	498	0.70	0.55	0.80	1.62	1.48	1.77
Ramucirumab (25)	Thrombosis	25	0.76	0.09	1.23	1.70	1.14	2.51
Cabozantinib (15)	Thrombosis	136	0.70	0.42	0.91	1.63	1.37	1.93
Bevacizumab (106)	Thrombosis in device	26	0.92	0.26	1.38	1.89	1.28	2.78
Aflibercept (47)	Thrombosis in device	5	2.14	0.58	3.13	4.42	1.84	10.62
Bevacizumab (106)	Thrombotic microangiopathy	188	2.42	2.18	2.59	5.34	4.62	6.18
Ramucirumab (25)	Thrombotic microangiopathy	21	3.32	2.59	3.84	10.01	6.52	15.38
Sunitinib (27)	Thrombotic microangiopathy	32	0.85	0.26	1.27	1.80	1.27	2.55
Sorafenib (37)	Thrombotic microangiopathy	27	1.67	1.03	2.13	3.19	2.18	4.65
Bevacizumab (106)	Thrombotic thrombocytopenic purpura	41	1.68	1.16	2.05	3.21	2.35	4.37
Ramucirumab (25)	Thrombotic thrombocytopenic purpura	6	2.58	1.16	3.49	5.97	2.68	13.31
Sunitinib (27)	Thrombotic thrombocytopenic purpura	15	1.19	0.32	1.80	2.29	1.38	3.80
Nintedanib (38)	Thrombotic thrombocytopenic purpura	9	1.22	0.08	1.98	2.33	1.21	4.48
Bevacizumab (106)	Transient ischaemic attack	183	0.68	0.43	0.85	1.60	1.38	1.85
Ramucirumab (25)	Transient ischaemic attack	16	1.48	0.64	2.06	2.79	1.71	4.56
Aflibercept (47)	Transient ischaemic attack	30	2.16	1.56	2.60	4.48	3.13	6.42
Lenvatinib (35)	Transient ischaemic attack	51	0.88	0.41	1.21	1.84	1.39	2.42
Nintedanib (38)	Transient ischaemic attack	92	1.47	1.12	1.72	2.77	2.26	3.40
Pazopanib (39)	Transient ischaemic attack	53	0.64	0.18	0.96	1.56	1.19	2.04
Apatinib (domestic) (35)	Tricuspid valve incompetence	8	2.52	1.31	3.33	5.75	2.87	11.52
Bevacizumab (106)	Troponin I increased	14	1.29	0.38	1.91	2.44	1.44	4.14
Aflibercept (47)	Troponin increased	8	2.27	1.05	3.07	4.81	2.40	9.63
Pazopanib (39)	Troponin increased	26	1.86	1.20	2.32	3.63	2.46	5.33
Axitinib (24)	Troponin increased	19	2.32	1.55	2.86	5.00	3.19	7.85
Bevacizumab (106)	Troponin T increased	10	1.18	0.10	1.90	2.26	1.21	4.23
Bevacizumab (106)	Tumour embolism	5	2.13	0.57	3.11	4.37	1.78	10.72
Sunitinib (27)	Tumour embolism	4	2.35	0.58	3.43	5.10	1.88	13.84
Sorafenib (37)	Tumour embolism	15	4.52	3.65	5.12	22.89	13.26	39.50
Axitinib (24)	Tumour embolism	3	2.52	0.45	3.72	5.72	1.81	18.01
Regorafenib (23)	Tumour embolism	6	3.35	1.94	4.26	10.21	4.48	23.26
Bevacizumab (106)	Tumour thrombosis	6	2.30	0.89	3.22	4.94	2.17	11.24
Pazopanib (39)	Tumour thrombosis	3	2.24	0.17	3.44	4.72	1.50	14.85
Sorafenib (37)	Tumour thrombosis	24	5.15	4.47	5.63	35.48	22.63	55.63
Regorafenib (23)	Tumour thrombosis	4	2.80	1.03	3.88	6.96	2.56	18.86
Bevacizumab (106)	Vena cava embolism	3	2.17	0.10	3.38	4.51	1.39	14.64
Bevacizumab (106)	Vena cava thrombosis	30	2.22	1.61	2.65	4.65	3.23	6.70

Ramucirumab (25)	Vena cava thrombosis	4	2.52	0.75	3.59	5.72	2.14	15.28
Aflibercept (47)	Vena cava thrombosis	16	4.31	3.46	4.89	19.78	12.06	32.46
Sunitinib (27)	Vena cava thrombosis	14	2.03	1.13	2.65	4.08	2.41	6.92
Pazopanib (39)	Vena cava thrombosis	12	2.44	1.47	3.11	5.44	3.08	9.63
Bevacizumab (106)	Venoocclusive liver disease	108	2.59	2.27	2.82	6.03	4.97	7.31
Apatinib (domestic) (35)	Venoocclusive liver disease	4	1.79	0.03	2.87	3.47	1.30	9.25
Sorafenib (37)	Venous occlusion	6	1.55	0.14	2.46	2.93	1.31	6.54
Bevacizumab (106)	Venous thrombosis	125	2.98	2.69	3.20	7.90	6.60	9.47
Ramucirumab (25)	Venous thrombosis	5	2.16	0.60	3.15	4.48	1.86	10.77
Nintedanib (38)	Venous thrombosis	19	1.98	1.21	2.52	3.96	2.52	6.21
Apatinib (domestic) (35)	Venous thrombosis	5	2.19	0.63	3.18	4.58	1.90	11.01
Bevacizumab (106)	Venous thrombosis limb	78	3.17	2.79	3.44	8.97	7.13	11.29
Bevacizumab (106)	Ventricular dysfunction	11	1.05	0.02	1.74	2.07	1.14	3.75
Aflibercept (47)	Ventricular dysfunction	4	2.53	0.76	3.61	5.77	2.16	15.43
Pazopanib (39)	Ventricular dysfunction	7	1.91	0.60	2.76	3.75	1.78	7.89
Pazopanib (39)	Ventricular dyskinesia	3	2.35	0.28	3.56	5.09	1.61	16.10
Bevacizumab (106)	Ventricular fibrillation	91	1.34	0.99	1.59	2.53	2.05	3.11
Bevacizumab (106)	Ventricular hypertrophy	13	1.66	0.72	2.30	3.16	1.82	5.48
Pazopanib (39)	Ventricular hypokinesia	19	2.45	1.68	2.99	5.47	3.48	8.60
Sorafenib (37)	Ventricular hypokinesia	7	1.36	0.06	2.22	2.57	1.22	5.41
Regorafenib (23)	Ventricular hypokinesia	6	1.46	0.05	2.37	2.75	1.23	6.13
Apatinib (domestic) (35)	Ventricular hypokinesia	4	2.21	0.45	3.29	4.63	1.74	12.36
Bevacizumab (106)	Visual acuity reduced transiently	13	2.82	1.88	3.46	7.06	4.03	12.40
Aflibercept (47)	Visual acuity reduced transiently	3	2.60	0.53	3.81	6.06	1.94	18.95
