# Supplementary material for: Cardiovascular toxicity associated with angiogenesis inhibitors: A comprehensive pharmacovigilance analysis based on the FDA Adverse Event Reporting System database from 2014 to 2021
Source: Front Cardiovasc Med. 2022 Oct 13;9:988013. doi: 10.3389/fcvm.2022.988013 (PMC9606330; doi:10.3389/fcvm.2022.988013)
Supplement: Supplementary file 5 [file Data_Sheet_5.PDF]

TABLE S13 The top 20 PTs with strongest signal value associated with angiogenesis inhibitors between intravenous mAbs and oral TKIs with anti-VEGF(R) activity

Preferred terms (PTs)	intravenous mAbs		oral TKIs	
	IC025	ROR025	IC025	ROR025
Arrhythmia supraventricular	2.43	5.65		
Ascites	2.74	6.77	2.26	4.85
Blood pressure diastolic increased			1.11	2.22
Blood pressure increased			2.04	4.14
Brachiocephalic vein thrombosis	2.09	4.65		
Cardiac ventricular scarring			0.98	1.97
Cardiopulmonary failure			1.48	2.87
Cerebral ischaemia	2.09	4.39		
Disseminated intravascular coagulation	2.19	4.64	1.27	2.46
Dyspnoea exertional			1.03	2.06
Ejection fraction decreased			1.29	2.49
Embolism	3.18	9.18		
Embolism arterial	2.51	5.92		
Embolism venous	2.88	7.51		
Hypertension	2.18	4.55	1.98	3.97
Hypertensive encephalopathy	2.27	5.16	1.43	2.86
Hypertensive urgency			1.13	2.38
Mesenteric vein thrombosis			1.16	2.36
Myocardial injury			1.25	2.62
Portal vein thrombosis	2.35	5.26	1.97	4.00
Pulmonary artery thrombosis	2.53	6.06		
Secondary hypertension			1.94	4.06
Splenic vein thrombosis			1.22	2.49
Superior vena cava syndrome	3.11	8.96	1.10	2.28
Systolic hypertension			1.43	2.88
Thrombophlebitis migrans	3.14	9.14		
Thrombotic microangiopathy	2.19	4.67		
Tumour embolism			3.03	8.25
Tumour thrombosis			3.24	9.49
Vena cava thrombosis	2.36	5.34		
Venoocclusive liver disease	2.15	4.57		
Venous thrombosis	2.65	6.43		
Venous thrombosis limb	2.75	6.94		
Visual acuity reduced transiently	2.17	4.85		

PT, preferred term; mAb, monoclonal antibody; TKI, tyrosine kinase inhibitors; VEGF(R), vascular endothelial growth factor (receptor); IC, information components; ROR, reporting odds ratio
