# Supplementary material for: Cardiovascular toxicity associated with angiogenesis inhibitors: A comprehensive pharmacovigilance analysis based on the FDA Adverse Event Reporting System database from 2014 to 2021
Source: Front Cardiovasc Med. 2022 Oct 13;9:988013. doi: 10.3389/fcvm.2022.988013 (PMC9606330; doi:10.3389/fcvm.2022.988013)
Supplement: Supplementary file 6 [file Data_Sheet_6.PDF]

TABLE S14 Comparisons of the PTs detected as signals according to IC_{025} associated with embolic and thrombotic events (SMQ) between intravenous mAbs and oral TKIs with anti-VEGF(R) activity

SMQs	Preferred terms (PTs)	IC_{025}	
		intravenous mAbs	oral TKIs
Embolic and thrombotic events, arterial (SMQ)	Acute coronary syndrome	1.53	0.27
	Aortic thrombosis	1.33	
	Arterial thrombosis	1.54	
	Blindness transient	1.04	
	Brachiocephalic vein thrombosis	2.09	
	Embolism arterial	2.51	
	Hepatic artery embolism		0.19
	Ischaemic stroke	0.30	
	Lacunar infarction	0.42	
	Mesenteric artery thrombosis	1.05	
	Myocardial necrosis	0.80	
	Pulmonary artery thrombosis	2.53	
	Pulmonary tumour thrombotic microangiopathy	1.26	
	Renal artery occlusion		0.09
	Retinal artery occlusion	0.92	
	Splenic artery thrombosis	1.95	
	Stress cardiomyopathy	0.60	
	Thrombotic microangiopathy	2.19	0.11
	Thrombotic thrombocytopenic purpura	1.27	
	Transient ischaemic attack	0.60	0.15
	Visual acuity reduced transiently	2.17	
Embolic and thrombotic events, venous (SMQ)	Axillary vein thrombosis	1.48	
	Brachiocephalic vein thrombosis	2.09	
	Deep vein thrombosis	1.36	
	Embolism venous	2.88	
	Jugular vein thrombosis	2.04	
	Mesenteric vein thrombosis	1.18	1.16
	Pelvic venous thrombosis	1.07	
	Portal vein occlusion		0.94
	Portal vein thrombosis	2.35	1.97
	Pulmonary embolism	1.57	0.41
	Pulmonary venous thrombosis		0.03
	Retinal vein occlusion	1.87	
	Renal vein thrombosis		0.30

	Retinal vein thrombosis		0.25
	Splenic vein thrombosis		1.22
	Subclavian vein thrombosis	1.60	
	Superior vena cava syndrome	3.11	1.10
	Thrombophlebitis	1.04	
	Thrombophlebitis migrans	3.14	
	Vena cava embolism	0.06	0.26
	Vena cava thrombosis	2.36	0.66
	Venoocclusive liver disease	2.15	
	Venous thrombosis	2.65	0.07
	Venous thrombosis limb	2.75	
Embolic and thrombotic events, vessel type unspecified and mixed arterial and venous (SMQ)	Cardiac ventricular thrombosis	1.79	0.56
	Catheter site thrombosis	1.34	
	Cerebellar infarction	0.13	
	Cerebral infarction	1.01	0.40
	Cerebral ischaemia	2.09	
	Cerebrovascular disorder	0.72	
	Choroidal infarction	1.14	
	Collateral circulation	1.08	
	Device related thrombosis	1.91	
	Disseminated intravascular coagulation	2.19	1.27
	Embolism	3.18	0.85
	Hemiparesis	1.42	
	Hemiplegia	0.94	
	Hepatic infarction	0.34	
	Intestinal infarction	0.41	
	Monoparesis	0.57	
	Splenic infarction	0.58	
	Thrombosis	0.53	
	Thrombosis in device	0.50	
	Thrombosis mesenteric vessel	0.30	
	Tumour embolism	0.88	3.03
	Tumour thrombosis	1.13	3.24

PT, preferred term; IC, information components; SMQ, the Standardized MedDRA Queries; mAb, monoclonal antibody; TKI, tyrosine kinase inhibitors; VEGF(R), vascular endothelial growth factor (receptor)
